# The effect of point-of-care ultrasound curriculum for nursing practitioners across different hospital levels

**DOI:** 10.1186/s12912-026-04328-1

**Published:** 2026-01-28

**Authors:** Joyce Tay, Meng-Che Wu, Wen-Yu Hu, Chien-Tai Huang, Cheng-Yi Wu, Wan-Ching Lien

**Affiliations:** 1 Department of Emergency Medicine, National Taiwan University Hospital and National Taiwan University, No.7, Chung-Shan South Road, Taipei, 100 Taiwan; 2 Department of Emergency Medicine, College of Medicine, National Taiwan University, Taipei, Taiwan; 3 Department of Nursing, National Taiwan University, Taipei, Taiwan; 4 Department of Emergency Medicine, National Taiwan University Hospital Hsin-Chu Branch, Hsin-Chu City, Taiwan

**Keywords:** Point-of-care ultrasound, Nursing practitioners, Objective structured clinical examination, Training, Learning

## Abstract

**Background:**

Literature describing nurse practitioners’ (NPs) experiences with point-of-care ultrasound (PoCUS) training and clinical use remains limited. This study aims to assess the effect of PoCUS training for NPs across different hospital levels.

**Methods:**

A prospective cohort study was conducted. The curriculum comprised 1-hour didactics followed by a 3-hour hands-on training session. Written examinations and objective structured clinical examinations (OSCEs) were administered immediately after training and at 3 months. Self-reported monthly PoCUS examination volumes during clinical practice after training were also collected.

**Results:**

One hundred and nineteen NPs across different hospital levels were recruited. They had better performance in both the written test and OSCE at the 3-month assessment compared to the immediate assessment. Following the curriculum, a greater proportion of NPs incorporated PoCUS into clinical practice, with abdominal ultrasound becoming the most used. Additionally, NPs working in settings without resident staff were significantly more likely to perform clinical PoCUS examinations (OR 4.43, 95% CI 1.57–12.47) after adjusting for covariates such as age, sex, postgraduate year, hospital level, department, prior experience, and the initial assessment.

**Conclusions:**

This focused PoCUS curriculum was associated with improved knowledge, skills and increased clinical use among NPs across different hospital levels, with greater uptake in teams without resident staff. However, interpretation is limited by short follow-up and self-reported use. Further studies with longer follow-up and direct assessment of clinical PoCUS performance are needed to better define training durability and the role of PoCUS in NP-led care.

**Trial registration:**

Registered at the ClinicalTrials.gov. (NCT 06543693).

**Supplementary Information:**

The online version contains supplementary material available at 10.1186/s12912-026-04328-1.

## Background

The nurse practitioner (NP)-staffed system model has emerged as an effective and practical solution to mitigate the challenges posed by resident shortages in healthcare settings. As the demand for healthcare services continues to rise, the NP-staffed model provides a sustainable alternative to traditional resident-staffed systems. Recent studies have consistently shown that the quality of care delivered by NP-staffed systems is comparable to that provided by resident-staffed systems, with no significant differences observed in patient outcomes across various care settings [1–3].

In Taiwan, the development of the NP program has played a pivotal role in integrating NPs into the healthcare workforce. Established in 2006, the NP program was designed to enable experienced registered nurses to enhance their clinical skills through standardized professional training. Upon completion of this rigorous training, candidates are required to pass a national examination to obtain NP licensure, qualifying them to take on advanced clinical roles within healthcare teams [4]. Numerous hospitals have successfully integrated NPs into their multidisciplinary teams, where they collaborate closely with physicians to provide comprehensive and high-quality patient care. Currently, there are approximately 15,000 registered NPs in Taiwan [5], of whom 40% work in university hospitals, 45% in community hospitals, and the remaining 15% in other healthcare settings.

Ultrasound (US) is a non-invasive diagnostic tool that does not involve ionizing radiation. Point-of-care ultrasound (PoCUS) is particularly suitable for bedside practice, serving as an excellent tool to complement patient assessment. Recently, the increasing application of PoCUS in nursing, such as in cardiac, pulmonary, and bladder assessments, as well as in the establishment of difficult intravenous access, have been reported [6–9].

There is limited literature describing the experiences of NPs with PoCUS training and its clinical application. Previous studies have been constrained by a small number of trainees and a predominance of participants from a single institution [10–15]. Consequently, it remains unclear whether NPs integrate PoCUS into their daily clinical practice and what factors may influence their usage following training.

This study aims to assess the effect of PoCUS training for NPs across different hospital levels.

## Methods

### Study setting

This prospective study was conducted at the National Taiwan University Hospital (NTUH) from August 2022 to December 2023. It was approved by the Institutional Review Board of the NTUH (202112120RINB) and registered at the ClinicalTrials.gov. (NCT 06543693). Informed consent was obtained from each participant.

All registered NPs were eligible for participation. Participants were recruited through posters on bulletin boards of the Taiwan Association of Nursing Practitioners. NPs aged 20 to 65 years who were interested in PoCUS and able to attend the training and complete all assessments were included. Those who were unable to attend the training or complete the assessments were excluded.

### PoCUS training

A PoCUS curriculum included a 1-hour didactics and a 3-hour hands-on training session with live healthy models. The teaching domains covered focused cardiac US, encompassing the subxiphoid cardiac view and inferior vena cava assessment; abdominal US, including liver, gall bladder, kidney, and urinary bladder assessment; intraperitoneal and intrapleural fluid assessment using the extended focused assessment with sonography for trauma (eFAST) protocol; and US-guided catheterization (Supplementary File 1), all with the consensus of the instructors. The instructor-to-participant ratio was less than 1:5. The instructors were expert sonographers, board-certified by the Taiwan Society of Ultrasound in Medicine, with over 10 years of experience in sonographic examinations.

After the curriculum, immediate assessments were conducted, including a written test and an objective structured clinical examination (OSCE) using a live model. The written test comprised 10 multiple-choice questions on ultrasound physics and image interpretation, with a total score of 100 points (10 points per question). The OSCE included standardized questions to evaluate image acquisition and quality in cardiac and abdominal US, intraperitoneal and intrapleural fluid assessment on liver models and US-guided catheterization on Blue Phantom™ (CAE Healthcare USA) and the assessment forms were shown in the Supplementary files 2–5. The global rating score using a Likert 5-point scale was given by the instructor on site. Another instructor, not involved in the training, also scored the performance through video review, with the participants’ faces covered. The scores given by the two instructors were then averaged. Also, the feedback on the curriculum (Supplementary File 6) was collected using a Likert 5-point scale (1 = strongly disagree; 2 = disagree; 3 = neutral; 4 = agree; 5 = strongly agree).

Three months after completing the curriculum, participants took a recall test, which included both a written exam and an OSCE, using the same format as the immediate assessments.

### Data collection

Before the curriculum, participants’ age, sex, post-graduate year, hospital level (university hospital, community hospital or clinic), department (medical or surgical), the structure of healthcare teams whether teams included residents or not, and prior US experience which included the number of US cases performed per month and the applications used were recorded.

There is evidence that OSCE global rating scores capture diverse levels of proficiencies better than checklists, and are easy for examiners to use [16]. During the immediate assessment, the written test scores and OSCE global rating scores were collected, as well as feedback on the curriculum. At the 3-month assessment, the written test scores and OSCE global rating scores were also collected, along with data on clinical sonographic examinations including the self-reported number of clinical PoCUS examinations performed per month and the applications used after the curriculum.

### Sample size estimation

According to the results of a previous study, mean skills test scores increased from 34.3% before the course to 51.0% at follow-up [17], indicating a meaningful improvement in performance. Based on an a priori power analysis assuming a two-sided significance level of 0.05, 80% statistical power, and a medium effect size (Cohen’s d = 0.5) [18], a minimum sample size of 32 participants was estimated to be sufficient for detecting a significant difference.

### Statistical analysis

The SAS software (SAS 9.4, Cary, North Carolina, USA) was employed for data analysis. Initially, we assessed the normality of continuous data using the Shapiro-Wilk test. For variables that were not normally distributed, data were summarized using medians and interquartile ranges (IQRs) and analyzed with Wilcoxon’s rank-sum test. Categorical variables were presented as counts and proportions, and statistical analyses were conducted using the Chi-square test or Fisher’s exact test, as appropriate.

To evaluate interrater reliability for the OSCE global rating scores between the two instructors, we used intraclass correlation (ICC) with a 95% confidence interval (CI). This approach ensured a robust measure of agreement between raters.

For assessing learning effects, we compared the NPs’ performance on written tests and OSCEs at both the immediate assessment and a follow-up at three months. We also compared the monthly number of NP-performed clinical sonographic examinations before and after the training curriculum.

Additionally, logistic regression models were utilized to identify factors associated with the likelihood of NPs performing clinical sonographic examinations following training, irrespective of the application. The covariates included age, sex, postgraduate year, hospital level, department, the structure of healthcare teams, prior US experience, averaged OSCE scores from the initial assessment. The odds ratio (OR) with a 95% CI was computed to evaluate the influence of these factors.

A p-value of less than 0.05 was considered statistically significant for all analyses.

## Results

### The characteristics of the participants

**Fig. 1:**
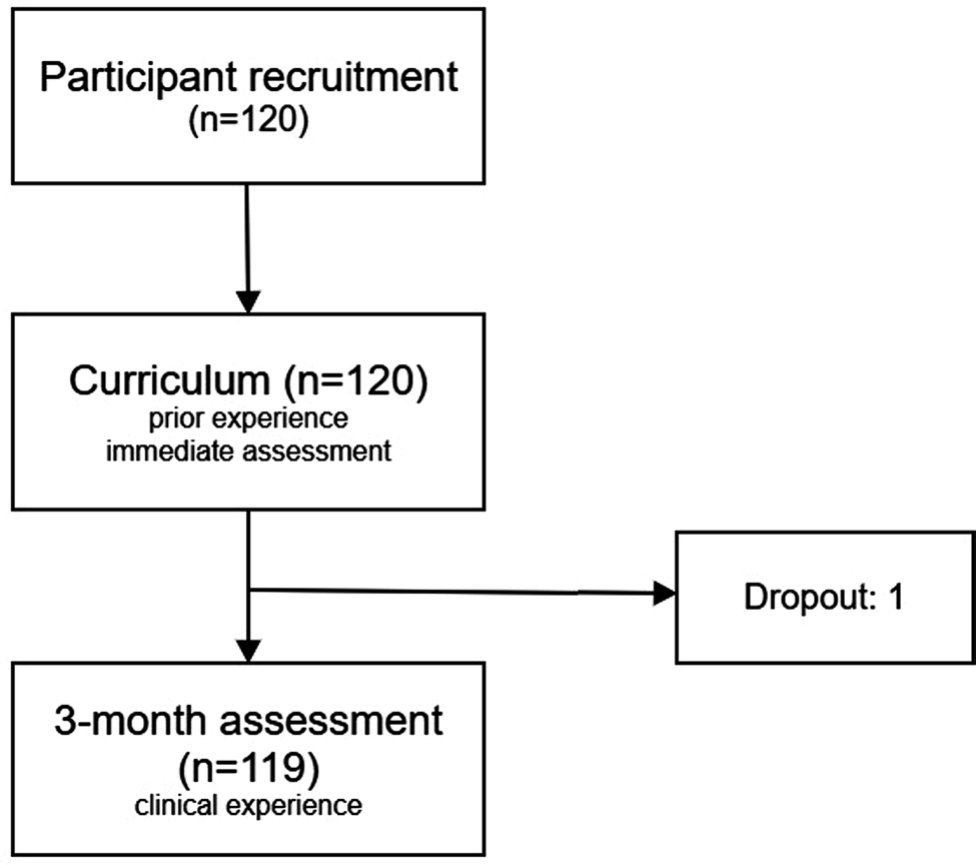
The study flowchart

A total of 120 NPs across different hospital levels were initially recruited. One NP did not complete the 3-month assessments and was therefore excluded, leaving 119 NPs in the final analysis (Fig. 1; Table 1). The median age of the participants was 42 years (IQR, 33–45), and 108 (91%) were female. More than half were employed in university hospitals (52%), followed by community hospitals (45%). Regarding departmental affiliation, 39% worked in medical departments and 61% in surgical departments. Most NPs (76%) were members of resident-staffed healthcare teams, and the majority (83%) had more than 10 years of post-graduate clinical experience. All the participants were novices, having performed fewer than 20 examinations prior to the study.

**Table 1:** The characteristics of the participants

Variables		Participants (*n* = 119)	
Age, years		42 (33–45)	
Female sex (%)		108 (91%)	
Working environment, n (%)			
University hospital		62 (52%)	
Community hospital		54 (45%)	
Clinic		3 (3%)	
Department, n (%) Medical		47 (39%)	
Surgical		72 (61%)	
Resident-staffed healthcare team, n (%)		90 (76%)	
Post-graduate year, n (%)			
1–5		7 (6%)	
6–10		13 (11%)	
>10		99 (83%)	
Previous ultrasound experience, n (%)			
No experience		57 (48%)	
1–20 cases		62 (52%)	

The Shapiro–Wilk test was used to assess the normality of continuous data. The results indicated that age, post-graduate year, prior ultrasound experience, written test scores, OSCE scores, the number of clinical sonographic examinations performed by NPs, and feedback scores were not normally distributed (all *p* < 0.0001). Accordingly, these variables were summarized using medians and interquartile ranges (IQRs) in the Tables and Supplementary File.

With respect to prior US experience, bladder assessment was the most commonly used application which 27 participants have performed, followed by intraperitoneal and intrapleural fluid assessment (14 participants). All the participants reported US machines were available at their hospitals/clinics. The feedback from participants was overwhelmingly positive (Supplementary Table 1).

### Interrater reliability of OSCE performance

There was strong interrater reliability in the OSCE global rating scores between the two instructors, with an ICC of 0.88 (95% CI, 0.70–0.93).

### The written test and OSCE performance of the participants after the training

Following the completion of the curriculum, NPs demonstrated improved performance in both the written test and the OSCE during the 3-month assessments compared to their immediate post-training results (Table 2). Additionally, there was a notable increase in the willingness of NPs to incorporate PoCUS into their clinical practice after the training.

**Table 2:** The performance of nursing practitioners during the immediate and 3-month assessments

Performance^*^	Immediate assessment	3-month assessment	*p*-Value
Written test score, points^†^	70 (60–90)	80 (70–90)	0.007
OSCE^‡^, global rating *score*^§^			
Heart and IVC^‡^	4 (3–4)	4 (4–5)	< 0.0001
Abdomen^a^	4 (4)	5 (4–5)	< 0.0001
Intraperitoneal/intrapleural fluid	4 (4)	5 (5)	< 0.0001
Catherization	3 (3–4)	5 (5)	< 0.0001

^*^Presented with medians (interquartile range)

^*†*^A total score of 100 points

^‡^OSCE, objective structured clinical examination; IVC, inferior vena cava

^*§*^Using a Likert 5-point scale

^*a*^Abdominal examinations comprising assessments of the liver, gallbladder, kidney, and urinary bladder

### Self-reported clinical performance after the training

After the curriculum, 63 NPs reported performing clinical sonographic examinations, of whom 42 worked in settings without resident staff (42/63 vs. 21/63, *p* = 0.014). We examined possible factors associated with NP-performed clinical PoCUS examinations using logistic regression models. Covariates included age, sex, post-graduate year, hospital level, department, healthcare team structure, prior ultrasound experience, and averaged OSCE scores from the initial assessment. NPs working in settings without resident staff were the only factor significantly associated with performing clinical PoCUS examinations (OR 4.43, 95% CI 1.57–12.47) after adjusting for the other covariates.

Self-reported data also revealed a distinct pattern in the clinical application of PoCUS (Table 3). Abdominal PoCUS—comprising assessment of the liver, gallbladder, kidney, and urinary bladder—emerged as the most frequently utilized application, followed by assessments for intraperitoneal and intrapleural fluid. Notably, many NPs reported using more than two different PoCUS applications in their clinical practice.

**Table 3:** The numbers of NPs performing clinical sonographic examinations before and after the curriculum, *as *self-reported by the participants

Application	Before the curriculum	After the curriculum	*p*-Value
Heart and IVC^*^, n (%)	1 (0.8%)	26 (23%)	< 0.0001
Abdomen^†^, n (%)	6 (5%)	53 (46%)	< 0.0001
Intraperitoneal/intrapleural fluid, n (%)	14 (12%)	38 (33%)	< 0.0001
Catherization, n (%)	10 (8%)	21 (18%)	0.026
≧ 2 applications, n (%)	0	48 (42%)^†^	< 0.0001

^*^IVC, inferior vena cava

^†^Abdominal examinations comprising assessments of the liver, gallbladder, kidney, and urinary bladder

^‡^After the curriculum, abdominal and intraperitoneal fluid assessments were the most commonly performed applications, reported by 35 of 48 participants

Based on clinical utilization, participants were classified into four groups: Group 1 included NPs who performed PoCUS both before and after the curriculum; Group 2 included those who performed PoCUS only after the curriculum; Group 3 included those who performed PoCUS only before the curriculum; and Group 4 included those who did not perform any sonographic examinations either before or after the curriculum (Table 4). No significant differences were observed in written test or OSCE scores among the four groups. However, it is noteworthy that a substantial proportion of NPs did not perform any PoCUS examinations after completing the training.

**Table 4:** The number of clinical sonographic examinations performed per month by nurse practitioners, self-reported before and after the curriculum

Application	Before the curriculum, *examinations* ^†^	After the curriculum, *examinations* ^†^	
Heart			
Group 1 (*n* = 1)	10	4	
Group 2 (*n* = 25)	-	2 (1–5)	
Group 3 (*n* = 0)	-	-	
Group 4 (*n* = 93)	0	0	
Abdomen^*^			
Group 1 (*n* = 2)	5, 10	5, 6	
Group 2 (*n* = 51)^*^	0	5 (2–10)	
Group 3 (*n* = 4)	2, 5, 5, 5	0	
Group 4 (*n* = 62)	0	0	
Intraperitoneal/intrapleural fluid			
Group 1 (*n* = 8)	5, 5, 8, 10, 10, 10, 10, 10	2, 5, 6, 10, 10, 25, 30, 100	
Group 2 (*n* = 30)	0	4 (2–10)	
Group 3 (*n* = 6)	6, 7, 10, 10, 10, 10	0	
Group 4 (*n* = 75)	0	0	
Catherization			
Group 1 (*n* = 4)	2, 2, 10, 16	1, 1, 3, 20	
Group 2 (*n* = 17)^*^	0	2 (1–3)	
Group 3 (*n* = 6)	2, 3, 5, 8, 10, 10	0	
Group 4 (*n* = 92)	0	0	

^*^Abdominal examinations comprising assessments of the liver, gallbladder, kidney, and urinary bladder

^†^ The number of examinations performed by each nurse practitioner (NP) was reported. For groups with more than 10 NPs, the data are presented as median and interquartile range (IQR)

## Discussion

In this study, we constructed an effective PoCUS curriculum for NPs across different hospital levels, comprising lecture and hands-on training. After finishing the curriculum, PoCUS knowledge and skills were maintained and further improved at the 3-month follow-up. In addition, a greater proportion of NPs reported performing PoCUS examinations in their clinical practice and expressed increased willingness to incorporate PoCUS into routine patient care. These findings collectively support the effectiveness of a focused PoCUS training program in addressing the educational needs of NPs. Most of the literature regarding POCUS education and training, and evaluation of efficacy primarily focuses on physicians. Studies examining the effectiveness of PoCUS training for NPs remain limited and are often constrained by small sample sizes [10, 11] or single-institution designs [14, 15]. To the best of our knowledge, our study includes the largest cohort of NPs across multiple hospital levels to date, thereby providing more robust evidence regarding the feasibility and effectiveness of PoCUS training in this population.

US is a non-invasive diagnostic modality without ionizing radiation and is widely used in daily clinical practice. PoCUS has gained broad acceptance across diverse clinical settings and has become an integral component of bedside assessment performed by various healthcare professionals [19–21]. Its ability to provide real-time imaging and immediate diagnostic information makes it particularly valuable for point-of-care decision-making. A recent review identified lack of formal training as the most significant barrier to PoCUS use among NPs and suggested that the most successful educational models include an introductory course followed by longitudinal reinforcement [22]. Our focused 4-hour curriculum, which combines structured instruction with supervised hands-on training, was designed to overcome common barriers such as limited time availability and inflexible schedules, thereby facilitating practical skill acquisition.

Our data demonstrated significant improvements in PoCUS knowledge and skills among novice NPs, along with an increased willingness to perform PoCUS in clinical practice. The curriculum was intentionally designed to meet practical clinical demands and facilitate the integration of PoCUS into everyday patient care. By focusing on essential applications and emphasizing hands-on experience, the program provides a practical and scalable framework that may be adapted to diverse healthcare settings.

However, the continued improvement in recall test scores at the 3-month follow-up should be interpreted with caution. This finding may reflect ongoing clinical exposure, self-directed learning, or exam-related preparation after training. In addition, test reactivity or a Hawthorne effect associated with repeated assessments may have contributed to the observed score increases. Future studies using objective performance measures or controlled study designs are needed to better distinguish true skill retention from testing-related effects.

In clinical practice, abdominal US was reported by NPs as the most commonly performed examination, whereas US-guided catheterization was the least frequently utilized. This disparity in usage may be attributed to various factors, including the routine nature of catheterization procedures for experienced NPs and evolving recommendations regarding the application of US in vascular access. A previous study reported that ultrasound-guided hemodialysis access, while beneficial as an adjunct, is not required for every cannulation [23]. This perspective aligns with clinical practice in Taiwan, where many NPs have extensive experience as registered nurses and routinely perform venipuncture and catheterization without US guidance. Their proficiency and confidence in these procedures likely contribute to lower adoption rates of US-guided catheterization. In contrast, the American Institute of Ultrasound in Medicine strongly recommends the use of US to assist peripheral vascular access when personnel are adequately trained [24]. This recommendation highlights the potential benefits of broader US integration, particularly in complex cases or patients with difficult vascular access, and suggests opportunities for further skill development among NPs.

Despite observed differences in US utilization across clinical applications, an important limitation of this study warrants emphasis. Specifically, clinical US images obtained by NPs after completion of the training program were not systematically collected. Consequently, although improvements in knowledge and performance in simulated assessments were demonstrated, the study was unable to directly evaluate post-training diagnostic accuracy, image acquisition quality, or the transfer of acquired skills to real-world clinical practice. Future studies should address this limitation by incorporating structured post-training assessment strategies, such as periodic image audits with expert review and workplace-based assessments to better capture real-world ultrasound performance.

While the NP participants provided overwhelmingly positive feedback on learning and utilizing PoCUS, a significant proportion did not continue to perform clinical PoCUS examinations after completing the curriculum. This finding indicates that despite their enthusiasm and willingness to incorporate PoCUS into their practice, multiple factors may limit the translation of training into routine practice. Commonly reported barriers include insufficient confidence, limited access to US machines, time constraints during clinical workflows, and lack of ongoing mentorship [22]. Addressing these barriers may be critical to sustaining PoCUS utilization following initial training.

One notable observation from the study is that NPs were particularly inclined to perform clinical PoCUS examinations within healthcare teams that did not include residents. Traditionally, diagnostic procedures such as PoCUS are often performed by residents within physician-led teams. However, the ongoing shortage of residents has necessitated the development of alternative staffing models. In response, NP-staffed teams, which include supervising physicians, NPs, and nurses, have emerged as a viable and effective solution to fill this gap [1–3]. Within these teams, NPs frequently assume first-line roles in patient assessment and initial decision-making. The integration of PoCUS into their clinical skill set enables timely, non-invasive diagnostic evaluation and enhances their capacity to manage patient care effectively. The integration of PoCUS into their skill set al.lows NPs to complement traditional patient evaluations, providing immediate and non-invasive diagnostic information that can enhance clinical decision-making. This expanded role not only enhances patient care but also underscores the versatility and adaptability of NPs in diverse healthcare settings.

Overall, our findings indicate that NPs working on teams without residents performed more PoCUS examinations than those working on teams with residents, suggesting that in the absence of residents, NPs may assume responsibilities traditionally performed by residents, including the use of advanced diagnostic tools such as PoCUS. These observations highlight the importance of providing structured PoCUS training and ongoing support to enable NPs to apply their skills effectively in clinical practice.

This study had several limitations. First, the participants were self-selected, as they voluntarily chose to join the study. This introduces the possibility of selection bias, where the participants may not be fully representative of the broader population of NPs, potentially skewing the results. Those who opted to participate may have been more motivated or already more inclined toward using PoCUS, which could influence the outcomes. However, it is important to note that our study included the largest cohort of participants in PoCUS training to date, enhancing the generalizability of the findings. Despite the potential for bias, the results are likely indicative of the most effective learning outcomes achievable through this curriculum. Second, although all participants reported access to US machines in their workplaces, clinical image data from post-training PoCUS examinations were not comprehensively collected. This incomplete capture of clinical images limited our ability to systematically evaluate image quality and may have influenced the overall assessment of training effectiveness. Furthermore, participants were recruited from multiple hospitals where US machines and default settings varied and were not standardized across sites. Such heterogeneity in equipment and settings may have contributed to variability in image quality and introduced additional measurement noise into the evaluation process. To partially mitigate these methodological constraints, a recall test was conducted in a controlled environment using standardized US equipment and settings, allowing learning outcomes to be assessed under uniform conditions. Nevertheless, future studies should aim to strengthen methodological rigor by implementing structured post-training assessment frameworks, such as standardized image archiving protocols, expert-led image review, and workplace-based evaluations, to more accurately capture real-world US performance. Third, the diagnostic accuracy of NP-performed PoCUS examinations was not directly evaluated in this study. In Taiwan, NPs do not have independent clinical US operation privileges and must perform these procedures under the supervision of attending physicians. As a result, the accuracy of PoCUS examinations reflects the judgments of the supervising physicians rather than the NPs’ interpretations. This limitation means that the study could not directly assess the diagnostic proficiency of NPs using PoCUS, which is a crucial aspect of evaluating the effectiveness of the training. Last, the study included a recall test conducted three months after the completion of the curriculum. Participants were informed of this follow-up test in advance, which may have influenced their preparation and performance, so-called the Hawthorne effect. While the recall test provided valuable insights into short-term retention of skills, it does not fully capture the extent of long-term skill retention or the sustained impact of the training program. Future research should explore these aspects to provide a more comprehensive understanding of how well NPs maintain and apply PoCUS skills over time.

## Conclusions

This study demonstrates that a focused PoCUS curriculum combining didactic instruction and hands-on training is associated with short-term improvements in US knowledge and image acquisition skills among novice NPs across different hospital levels. Assessments conducted three months after training showed that these competencies were maintained, with modest improvement, indicating short-term knowledge and skill retention following the intervention. In addition, participants reported increased willingness to incorporate PoCUS into their clinical practice, particularly in healthcare teams without residents. Abdominal US was the most frequently reported PoCUS application, reflecting its common role in routine clinical evaluation. While these findings suggest that a brief, focused training program can address immediate educational needs and support early adoption of PoCUS by NPs, the conclusions are limited by the short follow-up period and reliance on self-reported measures of clinical use. Further studies incorporating longer-term follow-up, objective performance metrics, and direct assessment of clinical impact are needed to better define the durability of training effects and the role of PoCUS in NP–led clinical care.

## Supplementary Information

Below is the link to the electronic supplementary material.

Supplementary Material 1

Supplementary Material 2

Supplementary Material 3

Supplementary Material 4

Supplementary Material 5

Supplementary Material 6

Supplementary Material 7

## Data Availability

All data analyzed during this study is included in this published article.

## Abbreviations

NP: Nurse practitioner
US: Ultrasound
PoCUS: Point-of-care ultrasound
NTUH: National Taiwan University Hospital
eFAST: Extended focused assessment with sonography for trauma
OSCE: Objective structured clinical examination
IQR: Interquartile range
ICC: Intraclass correlation
CI: Confidence interval
OR: Odds ratio

## References

[1] Liao MT, Chang HC, Chen CK, Cheng LY, Lin TT, Keng LT. Outcomes of daytime nurse practitioner-staffed versus resident-staffed nonsurgical intensive care units: A retrospective observational study. Aust Crit Care. 2022;35(6):630–5. 34857440 10.1016/j.aucc.2021.10.004

[2] Liu CF, Hebert PR, Douglas JH, Neely EL, Sulc CA, Reddy A, et al. Outcomes of primary care delivery by nurse practitioners: Utilization, cost, and quality of care. Health Serv Res. 2020;55(2):178–89. 31943190 10.1111/1475-6773.13246 PMC7080399

[3] Landsperger JS, Semler MW, Wang L, Byrne DW, Wheeler AP. Outcomes of nurse Practitioner-Delivered critical care: A prospective cohort study. Chest. 2016;149(5):1146–54. 26836900 10.1016/j.chest.2015.12.015 PMC4944779

[4] Tsay SL, Tsay SF, Ke CY, Chen CM, Tung HH. Analysis of nurse practitioner scope of practice in Taiwan using the longest policy cycle model. J Am Assoc Nurse Pract. 2019;31(3):198–205. 30550389 10.1097/JXX.0000000000000127

[5] Practitioners TAoN. Current Practice Status of Nurse Practitioners in Taiwan 2025. Available from: https://www.tnpa.org.tw/information/content.php?id=234%26t=26%26p=1%26c=Y.

[6] Gundersen GH, Norekval TM, Haug HH, Skjetne K, Kleinau JO, Graven T, et al. Adding point of care ultrasound to assess volume status in heart failure patients in a nurse-led outpatient clinic: A randomised study. Heart. 2016;102(1):29–34. 26438785 10.1136/heartjnl-2015-307798 PMC4717409

[7] Mumoli N, Vitale J, Giorgi-Pierfranceschi M, Cresci A, Cei M, Basile V, et al. Accuracy of Nurse-Performed lung ultrasound in patients with acute dyspnea: A prospective observational study. Medicine. 2016;95(9):e2925. 26945396 10.1097/MD.0000000000002925 PMC4782880

[8] Acuña J, Sorenson J, Gades A, Wyatt R, Stea N, Drachman M, et al. Handheld ultrasound: overcoming the challenge of difficult peripheral intravenous access in the emergency department. J Ultrasound Med. 2020;39(10):1985–91. 32333616 10.1002/jum.15303

[9] O’Farrell B, Vandervoort MK, Bisnaire D, Doyle-Pettypiece P, Koopman WJ, McEwan L. Evaluation of portable bladder ultrasound: accuracy and effect on nursing practice in an acute care neuroscience unit. J Neurosci Nurs. 2001;33(6):301–9. 11776712 10.1097/01376517-200112000-00004

[10] Yamada T, Ehara J, Funakoshi H, Endo K, Kitano Y. Effectiveness of point of care ultrasound (POCUS) simulation course and skills retention for Japanese nurse practitioners. BMC Nurs. 2023;22(1):21. 36691022 10.1186/s12912-023-01183-2 PMC9872333

[11] Sağlam C, Güllüpınar B, Karagöz A, Tandon S, Bilge O, Aykır M, et al. Verification of endotracheal tube position by emergency nurses using ultrasound: A repeated measures cadaver study. J Emerg Nurs. 2022;48(2):181–8. 35125290 10.1016/j.jen.2022.01.002

[12] Dornhofer K, Farhat A, Guan K, Parker E, Kong C, Kim D, et al. Evaluation of a point-of-care ultrasound curriculum taught by medical students for physicians, nurses, and midwives in rural Indonesia. J Clin Ultrasound. 2020;48(3):145–51. 31876301 10.1002/jcu.22809

[13] Graven T, Wahba A, Hammer AM, Sagen O, Olsen Ø, Skjetne K, et al. Focused ultrasound of the pleural cavities and the pericardium by nurses after cardiac surgery. Scand Cardiovasc J. 2015;49(1):56–63. 25611808 10.3109/14017431.2015.1009383 PMC4389761

[14] Kalam S, Selden N, Haycock K, Lowe T, Skaggs H, Dinh VA. Evaluating the effect of Nursing-Performed Point-of-Care ultrasound on septic emergency department patients. Cureus. 2023;15(6):e40519. 37461778 10.7759/cureus.40519 PMC10350309

[15] Steinwandel U, Gibson N, Towell A, Rippey JJR, Rosman J. Can a renal nurse assess fluid status using ultrasound on the inferior Vena cava? A cross-sectional interrater study. Hemodial Int. 2018;22(2):261–9. 29024379 10.1111/hdi.12606

[16] Nicholls D, Sweet L, Hyett J. Psychomotor skills in medical ultrasound imaging: an analysis of the core skill set. J Ultrasound Med. 2014;33(8):1349–52. 25063399 10.7863/ultra.33.8.1349

[17] Naito T, Hashizumi A, Sakai M, Yamamura E, Iwase M, Yamada K, et al. Sustained effects of bladder point-of-care ultrasound simulation exercise on nursing students: A prospective cohort study. BMC Med Educ. 2025;25(1):127. 39863895 10.1186/s12909-025-06729-3 PMC11765932

[18] Cohen J. Statistical power analysis for the behavioral sciences. 2nd ed. New York: Hillsdale, NJ: Lawrence Erlbaum Associates,; 1988.

[19] Popat A, Harikrishnan S, Seby N, Sen U, Patel SK, Mittal L, et al. Utilization of Point-of-Care ultrasound as an imaging modality in the emergency department: A systematic review and Meta-Analysis. Cureus. 2024;16(1):e52371. 38694948 10.7759/cureus.52371 PMC11062642

[20] Niblock F, Byun H, Jabbarpour Y. Point-of-Care ultrasound use by primary care physicians. J Am Board Fam Med. 2021;34(4):859–60. 34312281 10.3122/jabfm.2021.04.200619

[21] Reinoso-Párraga PP, González-Montalvo JI, Menéndez-Colino R, Perkisas S, Rivera-Deras I, Garmendia-Prieto B et al. Usefulness of point of care ultrasound in older adults: a multicentre study across different geriatric care settings in Spain and the United Kingdom. Age Ageing. 2024;53(7). 10.1093/ageing/afae165 39051145

[22] Resnyk J, Weichold A. Barriers to learning and performing point-of-care ultrasound (POCUS): an integrative review. J Prof Nurs. 2024;54:54–62. 39266108 10.1016/j.profnurs.2024.06.007

[23] Schoch M, Bennett PN, Currey J, Hutchinson AM. Nurses’ perceptions of point-of-care ultrasound for haemodialysis access assessment and guided cannulation: A qualitative study. J Clin Nurs. 2023;32(23–24):8116–25. 37661364 10.1111/jocn.16877

[24] AIUM. AIUM practice parameter for the use of ultrasound to guide vascular access procedures. J Ultrasound Med. 2019;38(3):E4–18. 30758889 10.1002/jum.14954

