# Supplementary material for: The effect of point-of-care ultrasound curriculum for nursing practitioners across different hospital levels
Source: BMC Nurs. 2026 Jan 28;25:168. doi: 10.1186/s12912-026-04328-1 (PMC12924278; doi:10.1186/s12912-026-04328-1)
Supplement: Supplementary file 2 — Supplementary Material 2 [file 12912_2026_4328_MOESM2_ESM.docx]

**Ultrasound assessement of the abdomen**

Name：_______________ Date：

|  | **Checklist** | **Fail** | **Pass** | **Success** |
| --- | --- | --- | --- | --- |
| 01 | Probe placement | Frequent readjustment of the probe position on the skin or only an inadequate field of view | Correctly position probe for proper field of view, but needs occasional readjustment | With minimal readjustment, the probe can be correctly positioned to obtain the proper view on the first try |
| 02 | Image acquisition | No recognizable structures | Partial visualization of the structures with the minimal criteria required for diagnosis met. | All structures were imaged with excellent image quality, fully supporting the diagnosis |
|  | Liver |  |  |  |
|  | Gall bladder |  |  |  |
|  | Kidney |  |  |  |
|  | Urinary bladder |  |  |  |

**Global Rating Score**

| **1** | **2** | **3** | **4** | **5** |
| --- | --- | --- | --- | --- |
| Unacceptable performance; multiple major inadequacies | Unacceptable performance; some major inadequacies | Acceptable performance; minor inadequacies | Acceptable performance | Exceptional performance; expert provider |

Assessor ______________
