# Supplementary material for: The effect of point-of-care ultrasound curriculum for nursing practitioners across different hospital levels
Source: BMC Nurs. 2026 Jan 28;25:168. doi: 10.1186/s12912-026-04328-1 (PMC12924278; doi:10.1186/s12912-026-04328-1)
Supplement: Supplementary file 4 — Supplementary Material 4 [file 12912_2026_4328_MOESM4_ESM.docx]

**Post-curriculum Feedback**

1. The curriculum is well-organized.

1=strongly disagree; 2=disagree; 3=neutral; 4=agree; 5=strongly agree.

2. The content of the curriculum is adequate.

1=strongly disagree; 2=disagree; 3=neutral; 4=agree; 5=strongly agree.

3. The content of the curriculum is adequate.

1=strongly disagree; 2=disagree; 3=neutral; 4=agree; 5=strongly agree.

4. The content is practical.

1=strongly disagree; 2=disagree; 3=neutral; 4=agree; 5=strongly agree.

5. The content meets my needs.

1=strongly disagree; 2=disagree; 3=neutral; 4=agree; 5=strongly agree.

6. This course helps enhance my job skills.

1=strongly disagree; 2=disagree; 3=neutral; 4=agree; 5=strongly agree.

7. I have confidence in performing sonographic examinations.

1=strongly disagree; 2=disagree; 3=neutral; 4=agree; 5=strongly agree.
