# Supplementary material for: The effect of point-of-care ultrasound curriculum for nursing practitioners across different hospital levels
Source: BMC Nurs. 2026 Jan 28;25:168. doi: 10.1186/s12912-026-04328-1 (PMC12924278; doi:10.1186/s12912-026-04328-1)
Supplement: Supplementary file 5 — Supplementary Material 5 [file 12912_2026_4328_MOESM5_ESM.docx]

**US-guided catherization**

Name：_______________ Date：

|  | **Checklist** | **Fail** | **Pass** | **Success** |
| --- | --- | --- | --- | --- |
| 01 | Ultrasound-guided localization | Many unnecessary probe/needle movements | Efficient time/motion but some unnecessary probe/needle movements | Efficient probe/needle movements |
| 02 | Visualization of needle | Needle tip not seen during entering, difficult to locate after entry | Needle tip not seen during entering but easily and quickly located after entry | Tip clearly seen during entering and after entry |
| 03 | Catheterization and fluid aspiration | Poor catheterization and no fluid is aspirated | Smooth catheterization and fluid is aspirated | Smooth and quick catheterization and fluid is aspirated smoothly |

**Global Rating Score**

| **1** | **2** | **3** | **4** | **5** |
| --- | --- | --- | --- | --- |
| Unacceptable performance; multiple major inadequacies | Unacceptable performance; some major inadequacies | Acceptable performance; minor inadequacies | Acceptable performance | Exceptional performance; expert provider |

Assessor ______________
