# Supplementary material for: The effect of point-of-care ultrasound curriculum for nursing practitioners across different hospital levels
Source: BMC Nurs. 2026 Jan 28;25:168. doi: 10.1186/s12912-026-04328-1 (PMC12924278; doi:10.1186/s12912-026-04328-1)
Supplement: Supplementary file 6 — Supplementary Material 6 [file 12912_2026_4328_MOESM6_ESM.docx]

Supplementary Table 1. The feedback of the participants after the curriculum.

| Item | Participants (n=119) |
| --- | --- |
| The curriculum is well-organized. | 5 (5) |
| The content of the curriculum is adequate. | 5 (5) |
| The content is practical. | 5 (5) |
| The content meets my needs. | 5 (5) |
| This course helps enhance my job skills | 5 (5) |
| I have confidence in performing sonographic examinations | 5 (4-5) |

^*^presented with median and interquartile ranges.
