# Supplementary material for: The effect of point-of-care ultrasound curriculum for nursing practitioners across different hospital levels
Source: BMC Nurs. 2026 Jan 28;25:168. doi: 10.1186/s12912-026-04328-1 (PMC12924278; doi:10.1186/s12912-026-04328-1)
Supplement: Supplementary file 7 — Supplementary Material 7 [file 12912_2026_4328_MOESM7_ESM.pdf]

POCUS for NPs

Agenda

- **Basic physics**
- **Core content**
  - Intraperitoneal/intrapleural fluid (eFAST)
  - Subxiphoid cardiac view, IVC
  - Abdomen: liver, GB, kidney, UB
  - Venous cannulation

重點式照護超音波(Point-Of-Care-Ultra Sound, POCUS)

- I-AIM
- I- Indication (適應症):想要進行掃描和回答的臨床問題?
- A-Acquisition (取得影像):如何掃描到最佳影像?該進行哪些掃描?
- I-Interpretation(影像判讀):得到的影像的臨床意義?可能的診斷?
- M-Medical decision making (臨床決策): 掃描完成的同時進行判讀,並決定是否可以確認或排除特定的疾病或回答臨床問題?是否需要進行其他檢查?

I-AIM: a novel model for teaching and performing focused sonography
David P Bahner 1, Daralee Hughes, Nelson A Royall

超音波的發射與接收

- 超音波透過「可傳導音波的介質或組織」，
- 當碰到不同密度的組織介面時，部份的能量會被**反射**回來，
- 這些反射回來的**音波**能量，再被探頭的**壓電晶體**接收到，轉換成**電能訊號**，變成「**灰白對比色調**」的影像，呈現在螢幕上。

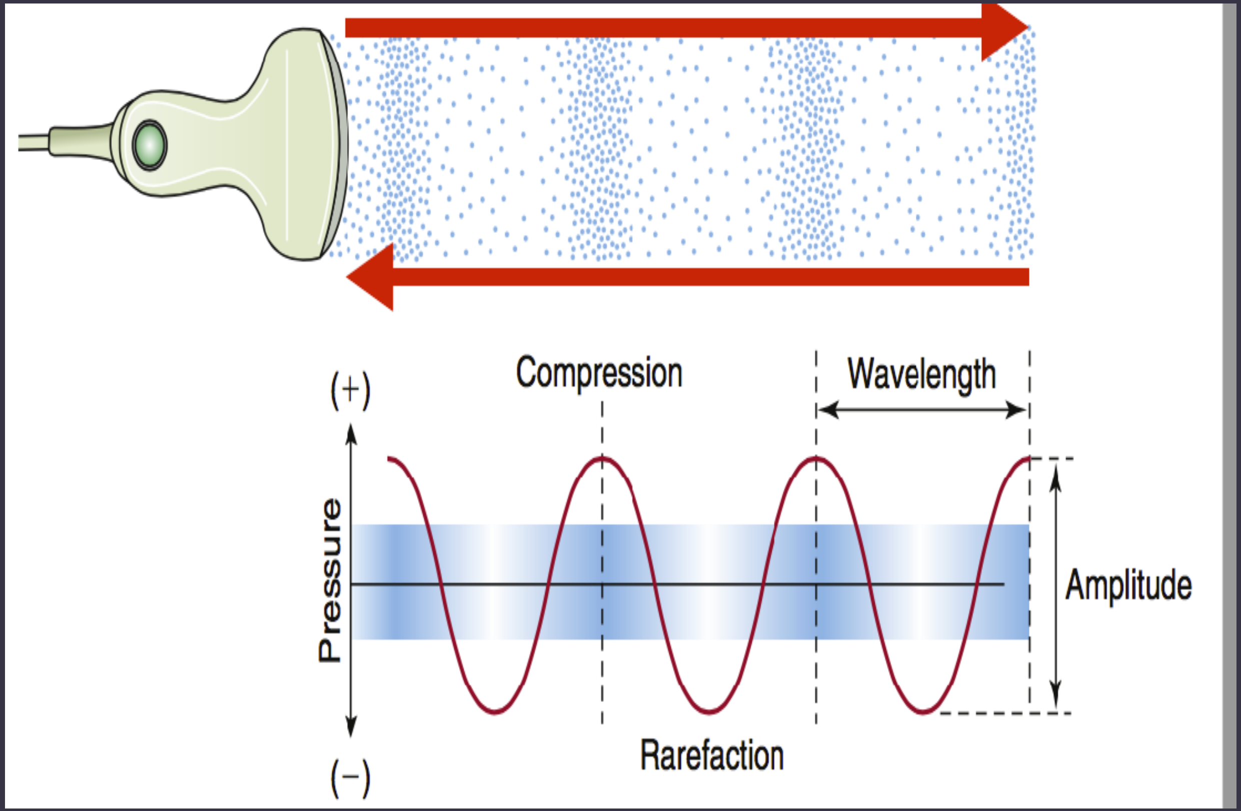

探頭的波長決定穿透力

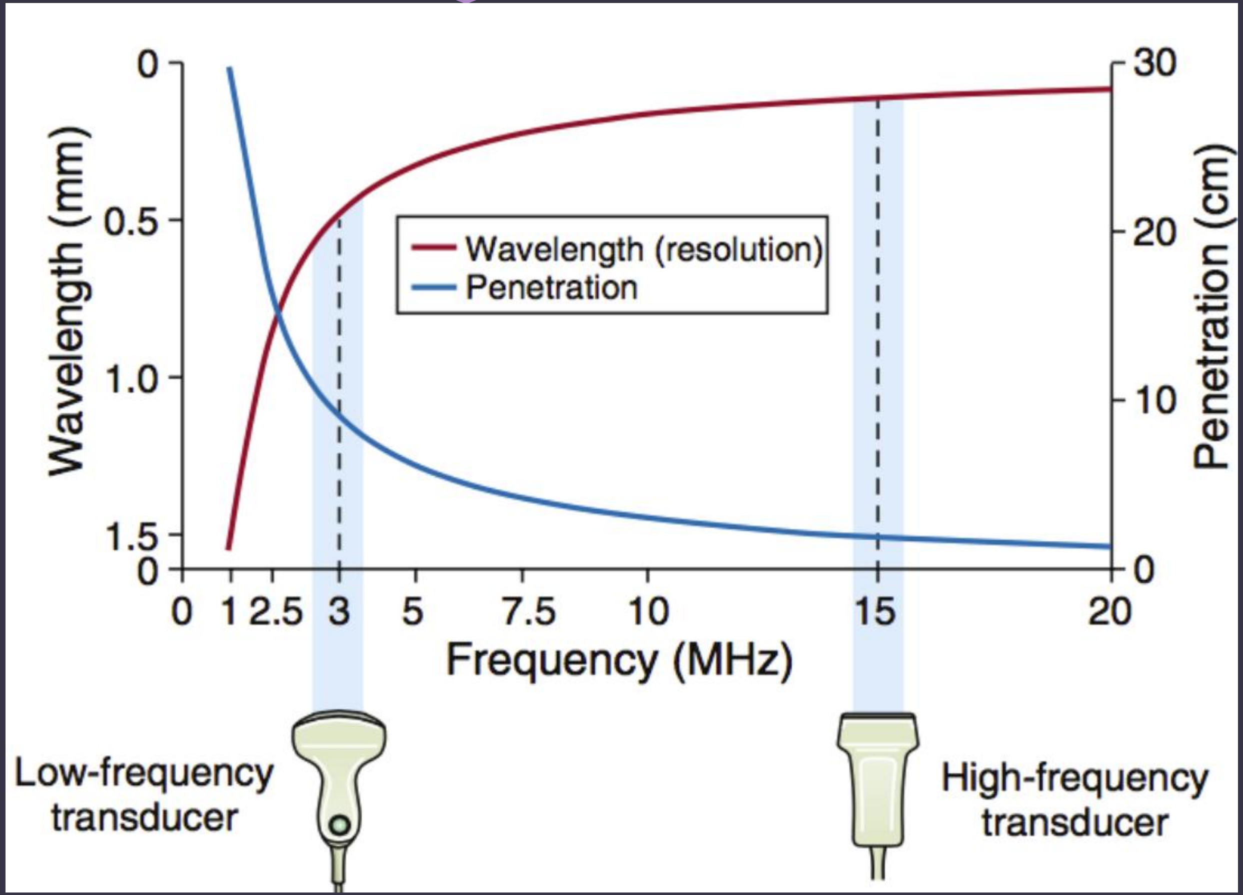

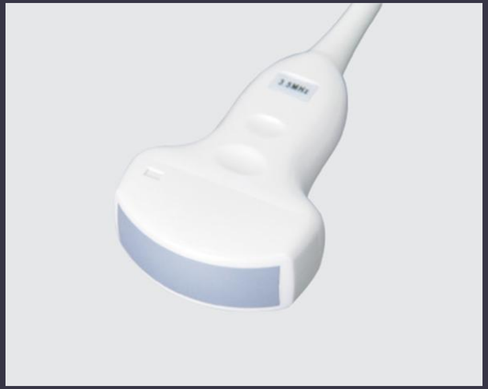

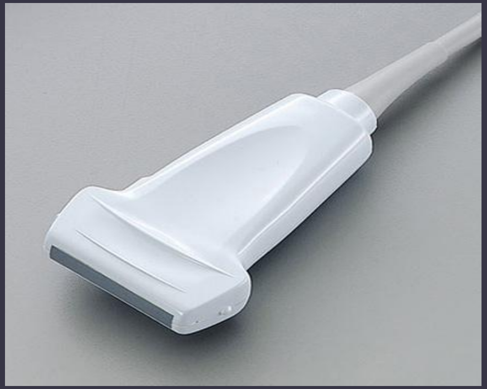

- Convex
- Abdomen
- 2-5 MHz

- Linear
- MSK
- 8-18 MHz

不同組織的衰減系數

Tissue	Attenuation coefficient	Ultrasound characteristics
Air	4500	Poor propagation, sound waves often scattered
Bone	870	Very echogenic (reflects most back, high attenuation)
Muscle	350	Echogenic (bright echo)
Liver/kidney	90	Echogenic (less bright)
Fat	60	Hypoechoic (dark echo)
Blood	9	Hypoechoic (very dark echo)
Fluid	6	Hypoechoic (very dark echo, low attenuation)

衰減系數（英語：attenuation coefficient）通常是指某些物理量例如光子、聲波、電子、粒子的數量或能量等等，**在物體中單一方向行進貫穿的難易程度。**

螢幕上「**灰白對比色調**」影像的強度，是由反射回來的超音波能量大小來決定

- 反射回來的能量越大，在螢幕上呈現的亮點越白

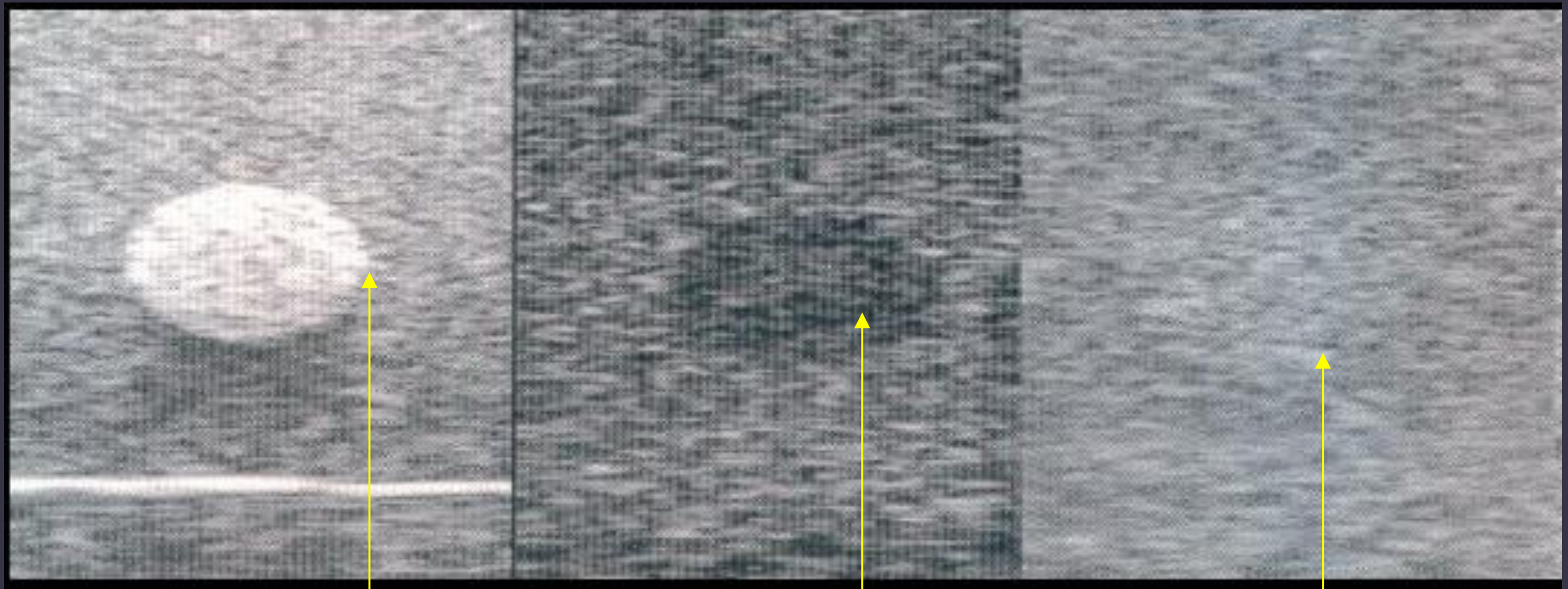

Hyperechoic
高回音
結石

Hypoechoic
低回音
血液

Isoechoic
等回音

螢幕上灰白影像點的深淺位置，
是由超音波射出後，又反射回來的時間所決定。

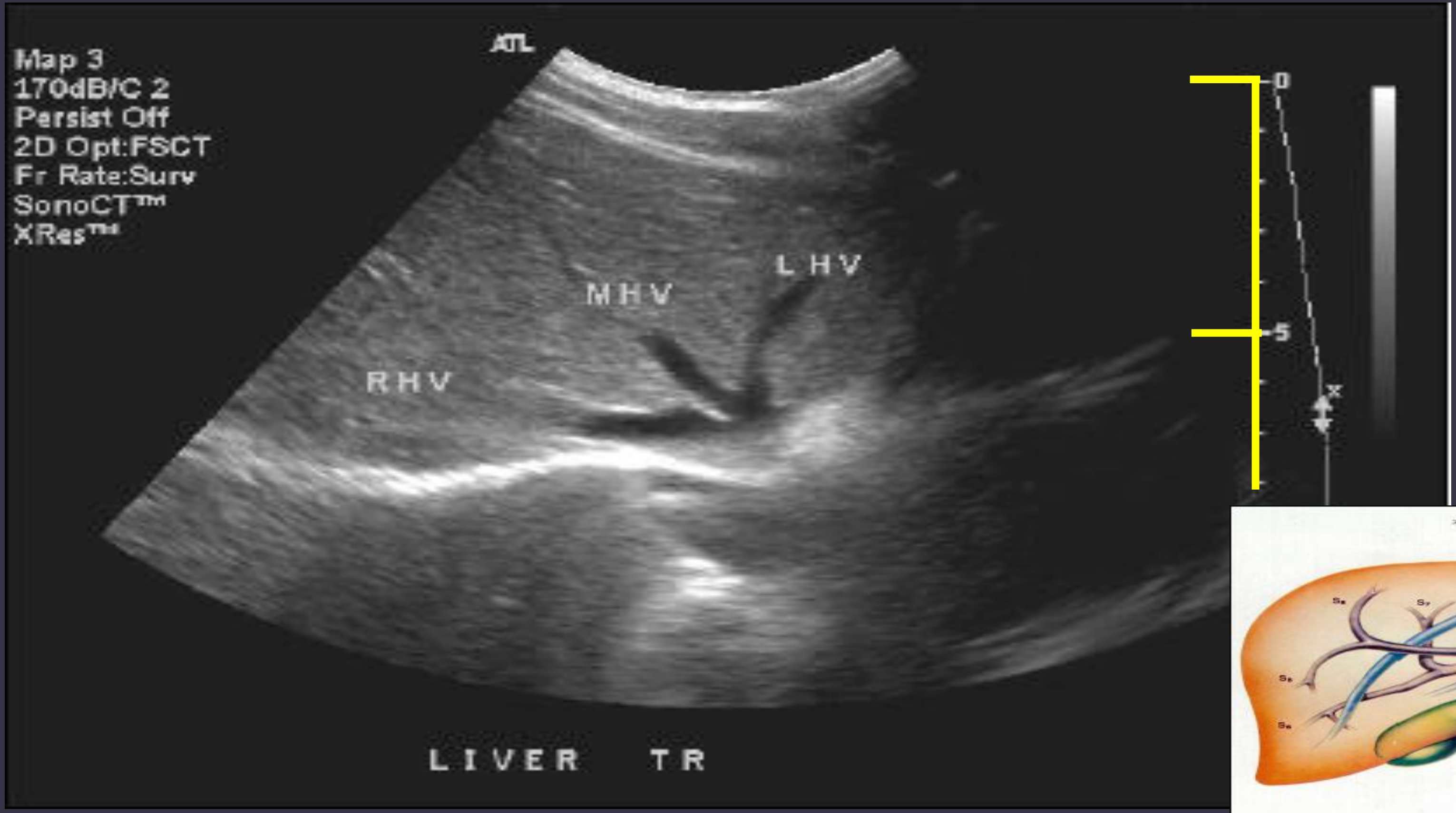

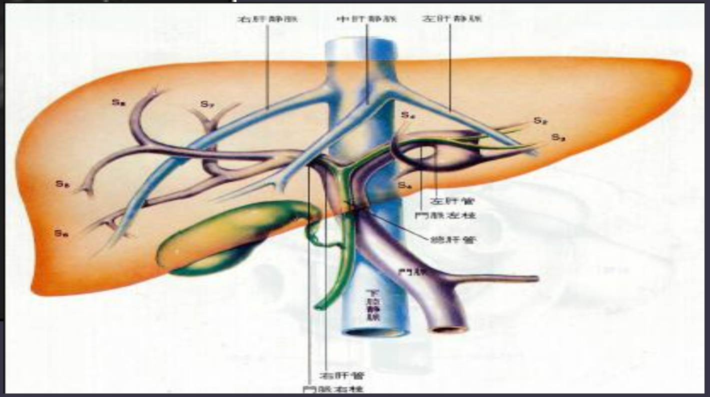

水是最好的朋友; 空氣是最大的敵人

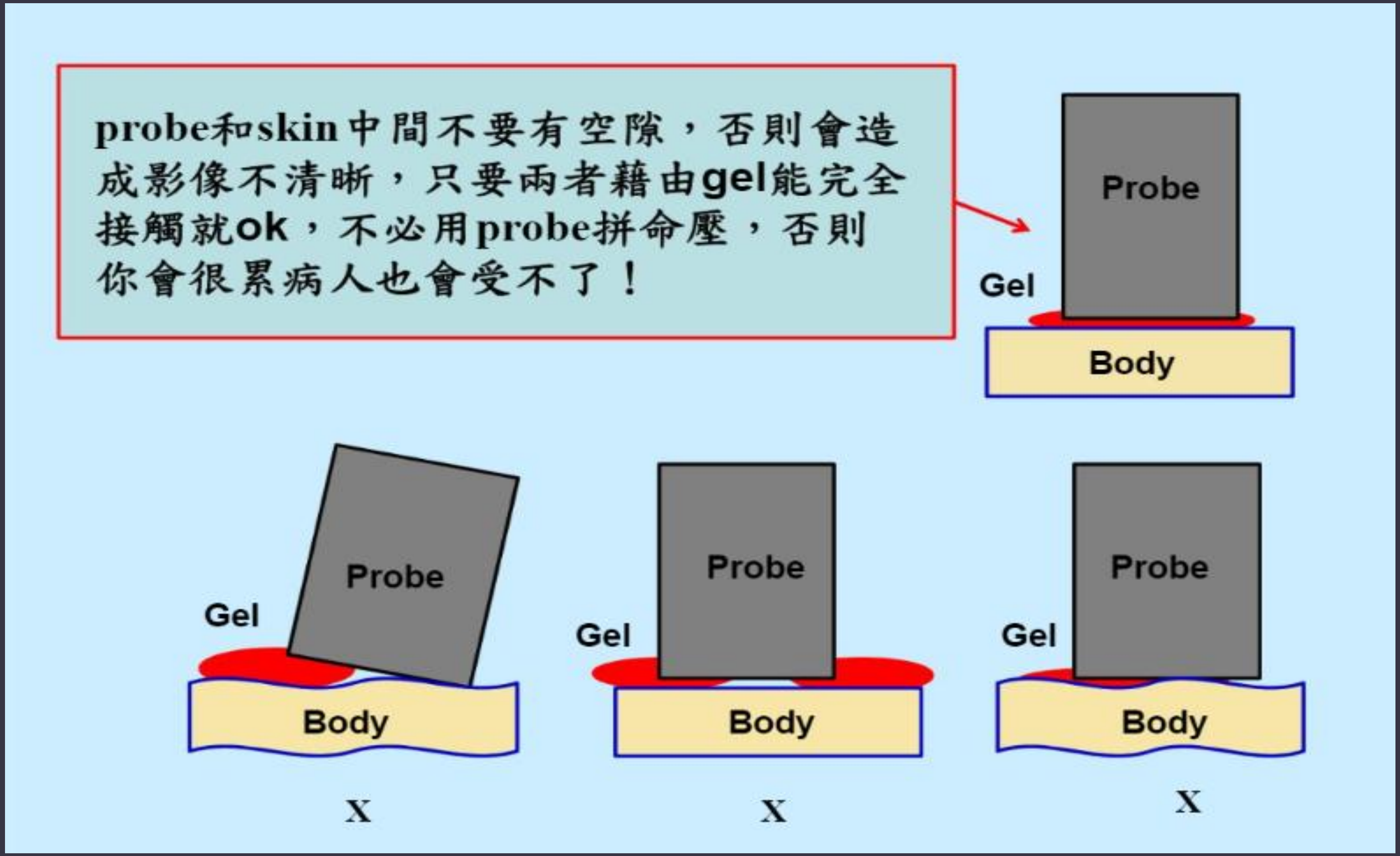

探頭必須垂直於掃描目標

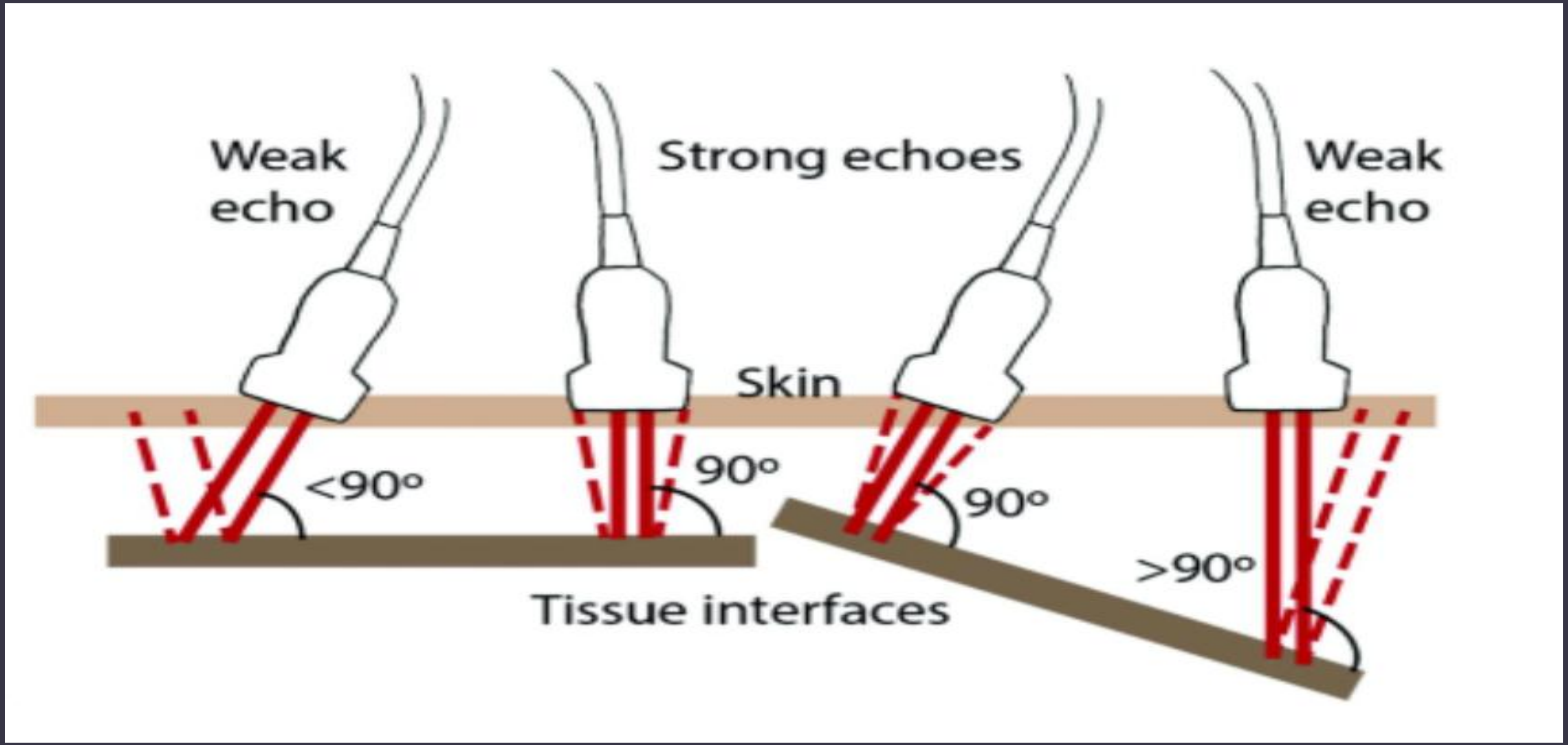

Acoustic shadow (回音後陰影)

- 超音波接觸到物體表面後，最基本的變化是**反射**。
- 當超音波的介質間密度相差越大，超音波反射程度就越多，而骨頭相對於身體其他組織來說結構較緻密，所以超音波會由表面反射掉，所以會在骨頭後方形成像影子一樣的artifact（同樣的情形一樣會出現在結石）

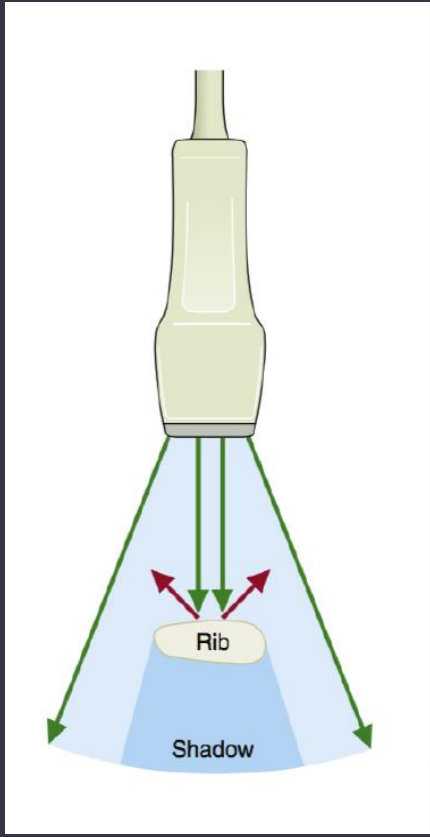

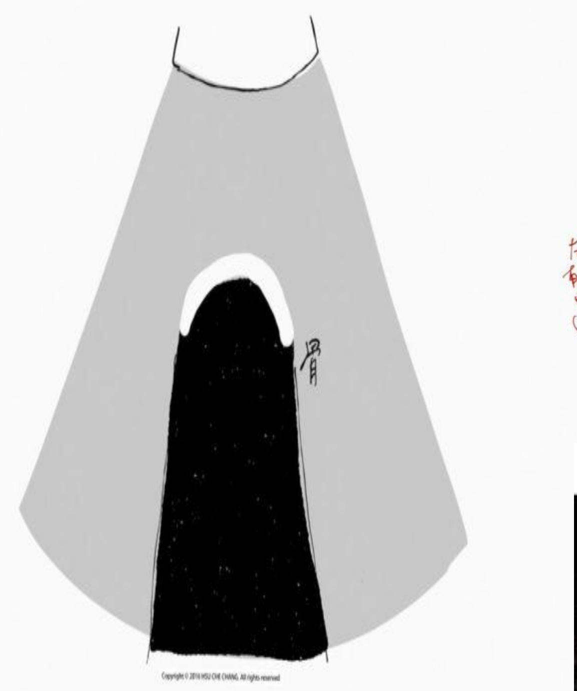

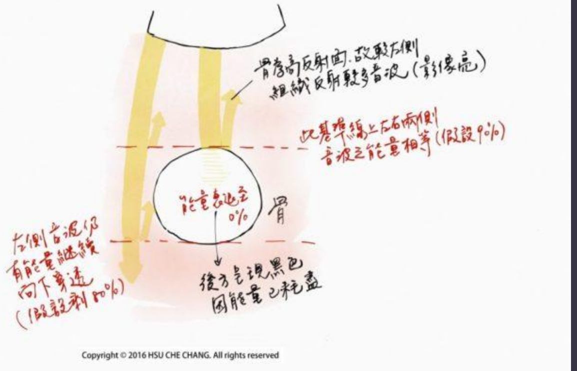

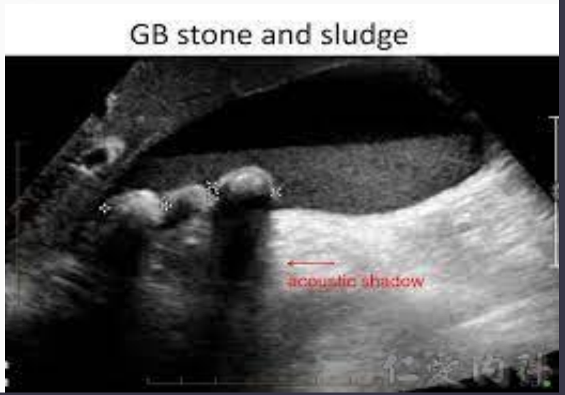

Posterior enhancement

- 指含液體的組織(如cyst, vessel等)後方因為聲波加成效果而會顯得更清楚(“白”/“亮”)一些

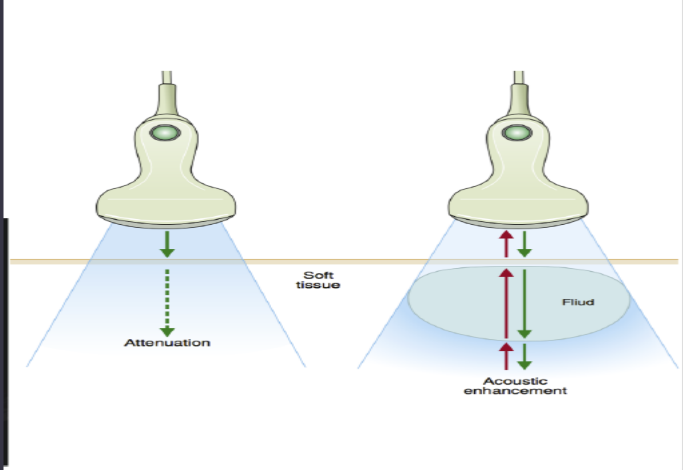

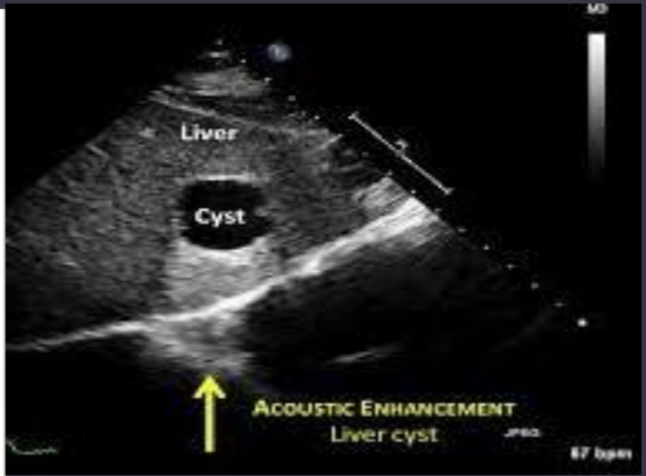

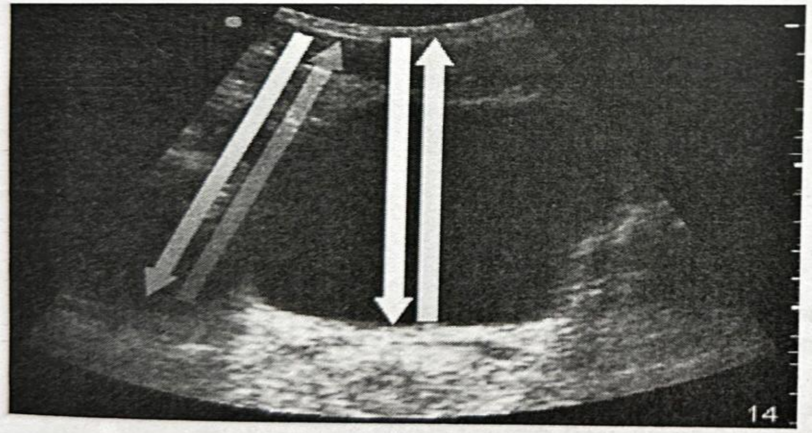

Figure 1.29
Posterior acoustic enhancement posterior to the bladder. The same acoustic energy (represented by the brightness of the arrows) enters the tissue. After significant attenuation (left pair) or no attenuation (right pair) the beams return with different amounts of energy. These are assigned different brightness values by the ultrasound machine.

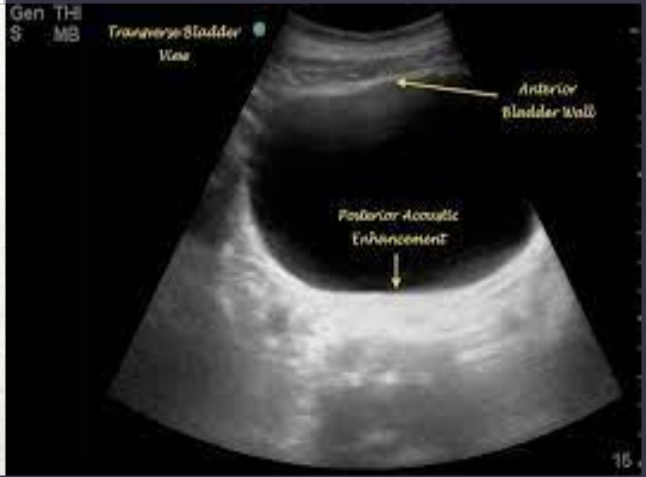

Core content

- Intraperitoneal/intrapleural fluid (eFAST)
- Subxiphoid cardiac view, IVC
- Abdomen: liver, GB, kidney, UB
- Venous cannulation

Intraperitoneal/intrapleural fluid (eFAST)

Extended - **F**ocused **A**ssessment with **S**onography for **T**rauma

- Focused Questions:
  - Is there free fluid/blood in the abdomen?
  - Is there fluid/blood in the pericardium?
  - Is there fluid/blood in the thorax?
  - Is there pneumothorax?

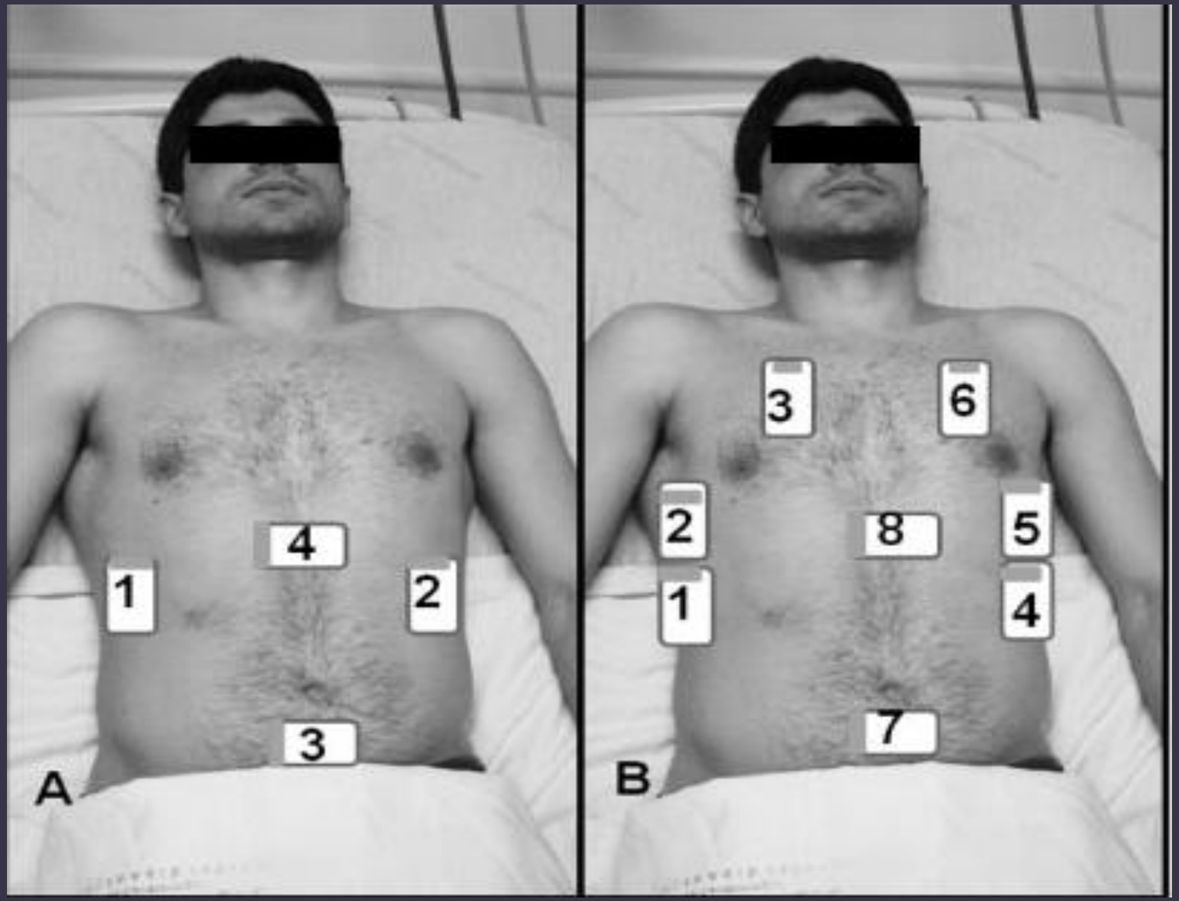

Advantages

- Rapid
- Repeat
- Non-invasive
- Portable
- No radiation or contrast

Disadvantages

- Depends on operator ➔ practice!!
- Difficult to distinguish ➔ practice!!
- Type of fluid
- Solid organ injury
- Difficulty for evaluate retroperitoneum
- Difficult in the obese patient
- Subcutaneous emphysema

RUQ view
Morison's pouch

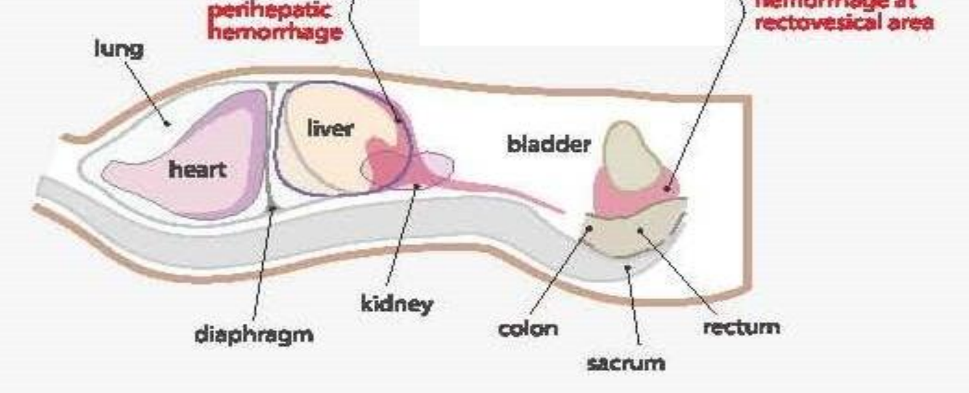

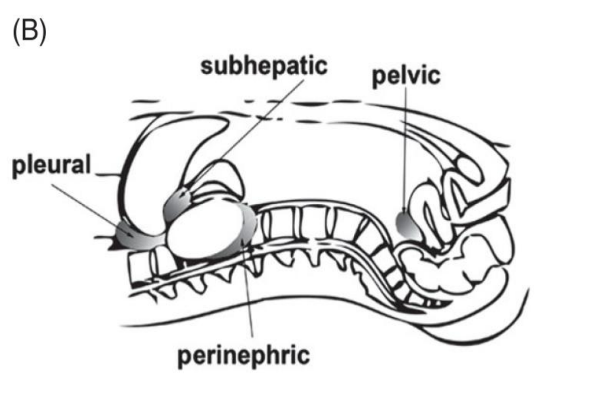

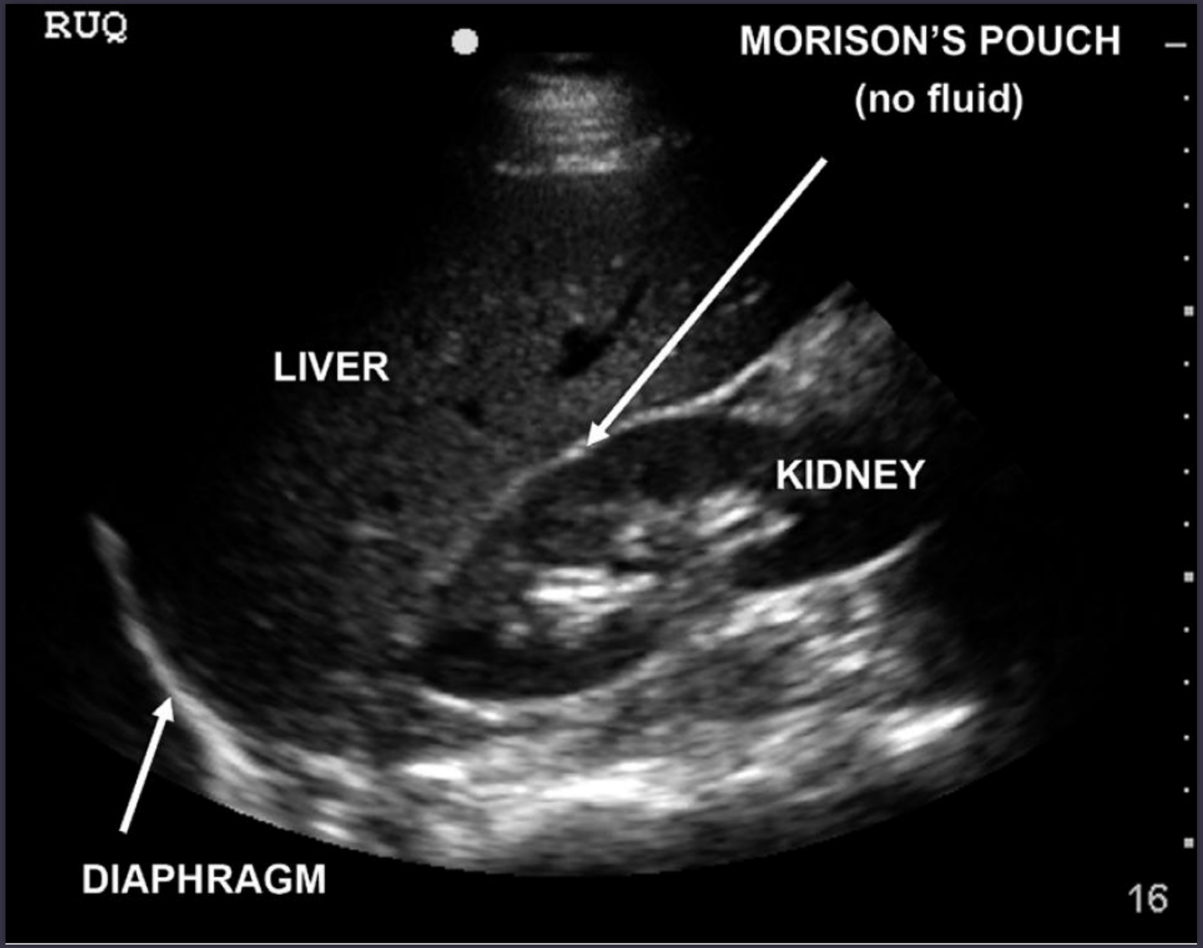

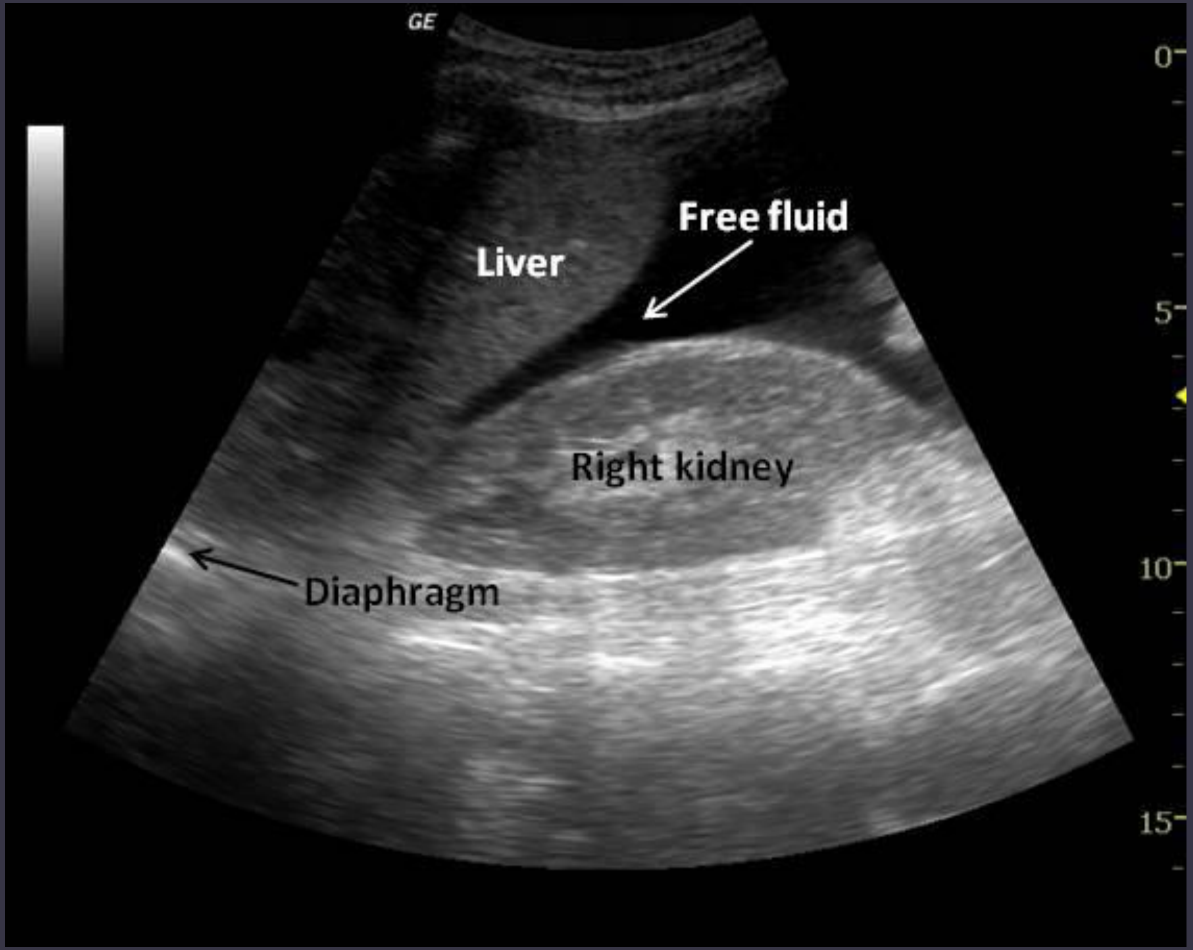

LUQ view
Splenorenal recess

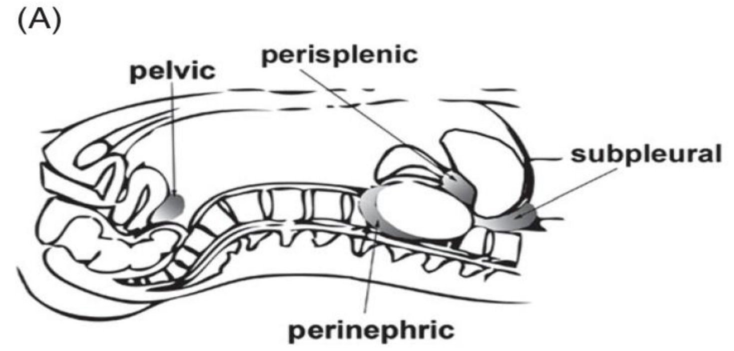

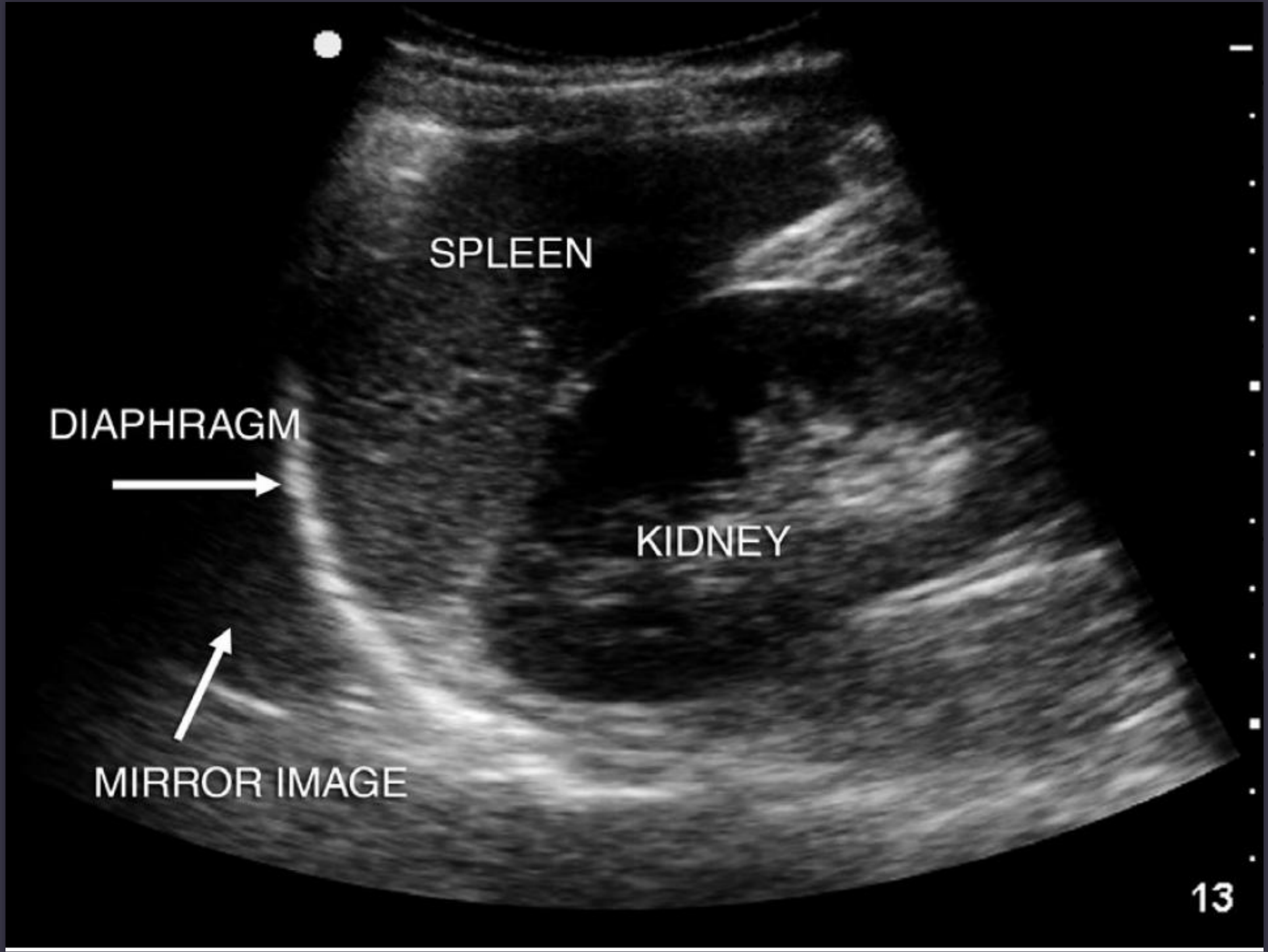

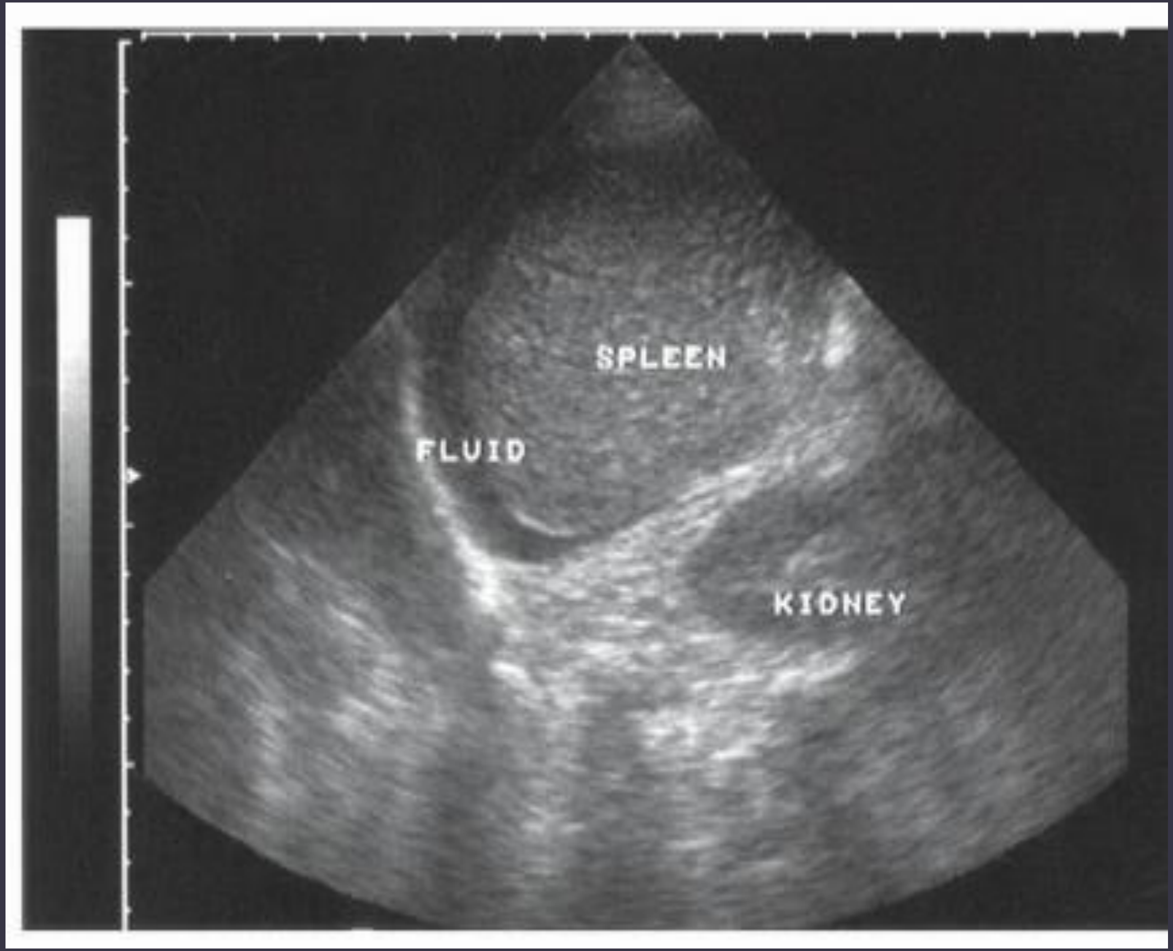

Pelvis view

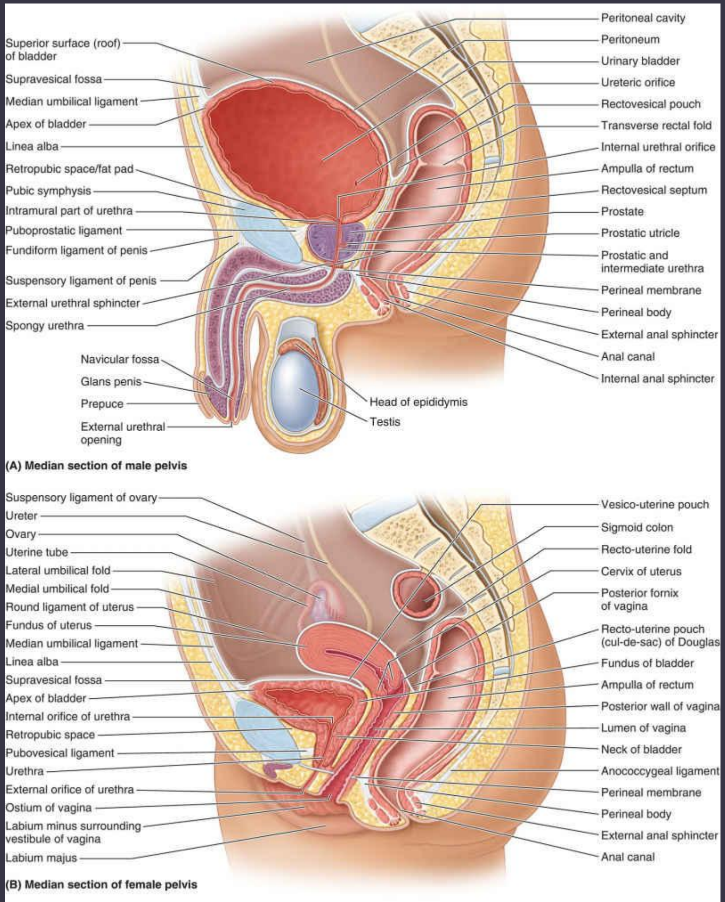

Superior surface (roof) of bladder
Supravesical fossa
Median umbilical ligament
Apex of bladder
Linea alba
Retropubic space/fat pad
Pubic symphysis
Intramural part of urethra
Puboprostatic ligament
Fundiform ligament of penis
Suspensory ligament of penis
External urethral sphincter
Spongy urethra
Navicular fossa
Glans penis
Prepuce
External urethral opening
Peritoneal cavity
Peritoneum
Urinary bladder
Ureteric orifice
Rectovesical pouch
Transverse rectal fold
Internal urethral orifice
Ampulla of rectum
Rectovesical septum
Prostate
Prostatic utricle
Prostatic and intermediate urethra
Perineal membrane
Perineal body
External anal sphincter
Anal canal
Internal anal sphincter
Head of epididymis
Testis
(A) Median section of male pelvis
Suspensory ligament of ovary
Ureter
Ovary
Uterine tube
Lateral umbilical fold
Medial umbilical fold
Round ligament of uterus
Fundus of uterus
Median umbilical ligament
Linea alba
Supravesical fossa
Apex of bladder
Internal orifice of urethra
Retropubic space
Pubovesical ligament
Urethra
External orifice of urethra
Ostium of vagina
Labium minus surrounding vestibule of vagina
Labium majus
Vesico-uterine pouch
Sigmoid colon
Recto-uterine fold
Cervix of uterus
Posterior fornix of vagina
Recto-uterine pouch (cul-de-sac) of Douglas
Fundus of bladder
Ampulla of rectum
Posterior wall of vagina
Lumen of vagina
Neck of bladder
Anococcygeal ligament
Perineal membrane
Perineal body
External anal sphincter
Anal canal
(B) Median section of female pelvis

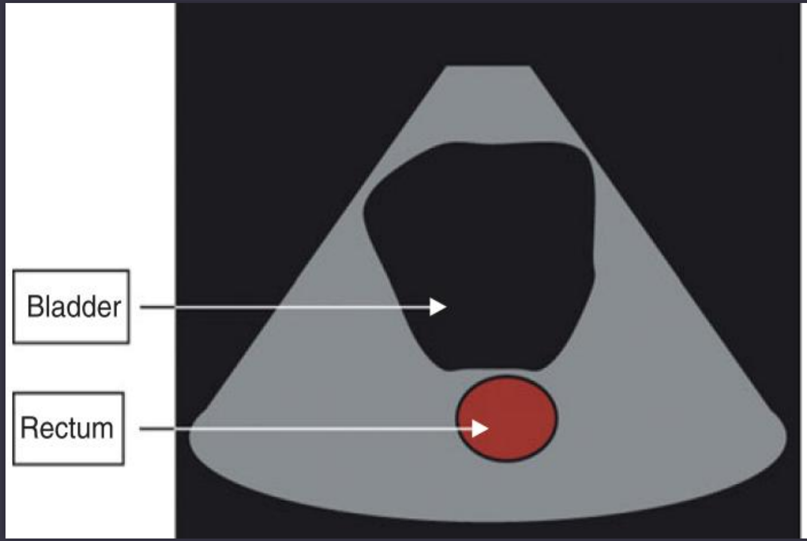

Bladder
Rectum

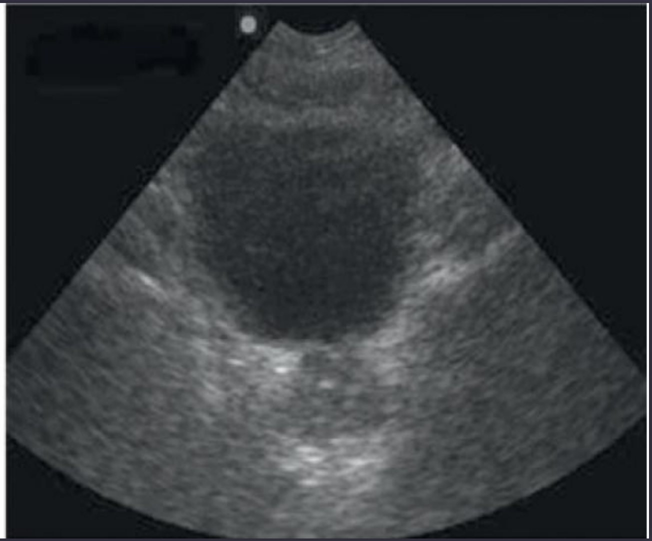

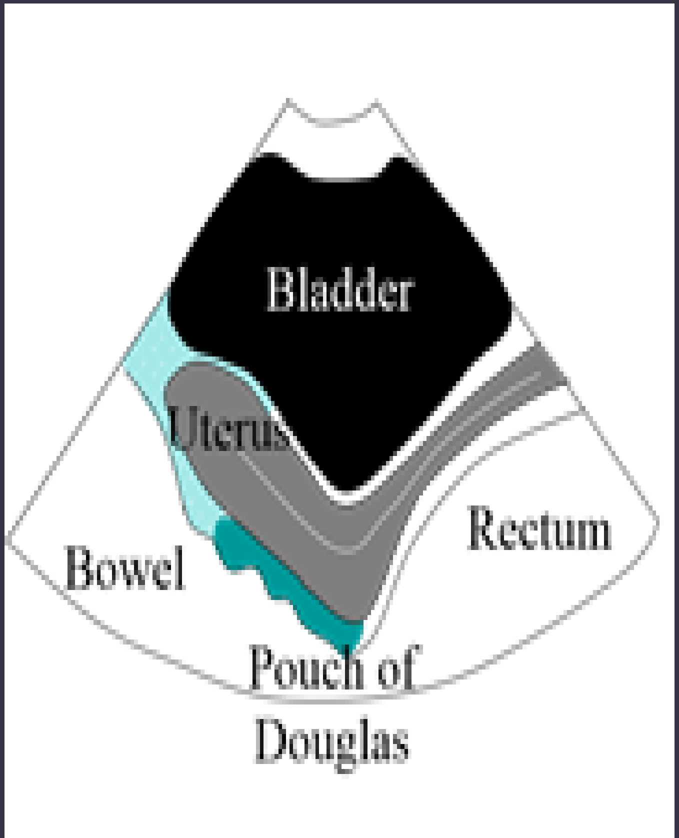

Bladder
Uterus
Bowel
Rectum
Pouch of Douglas

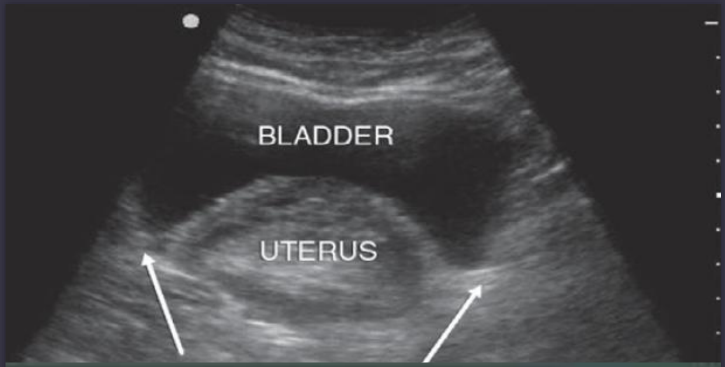

BLADDER
UTERUS

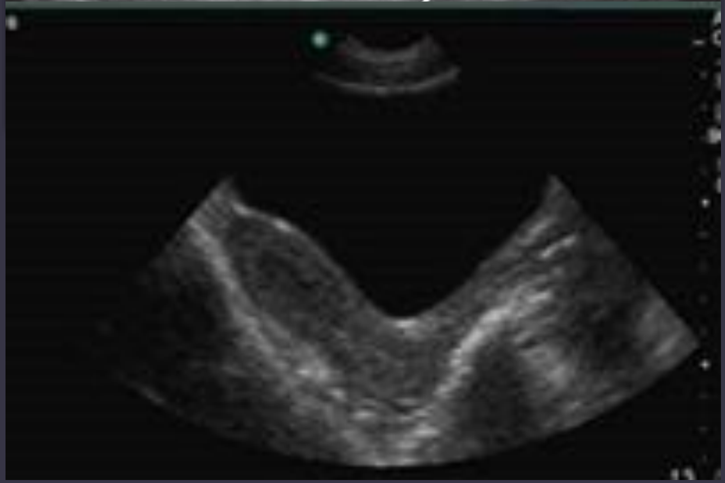

Pelvis view

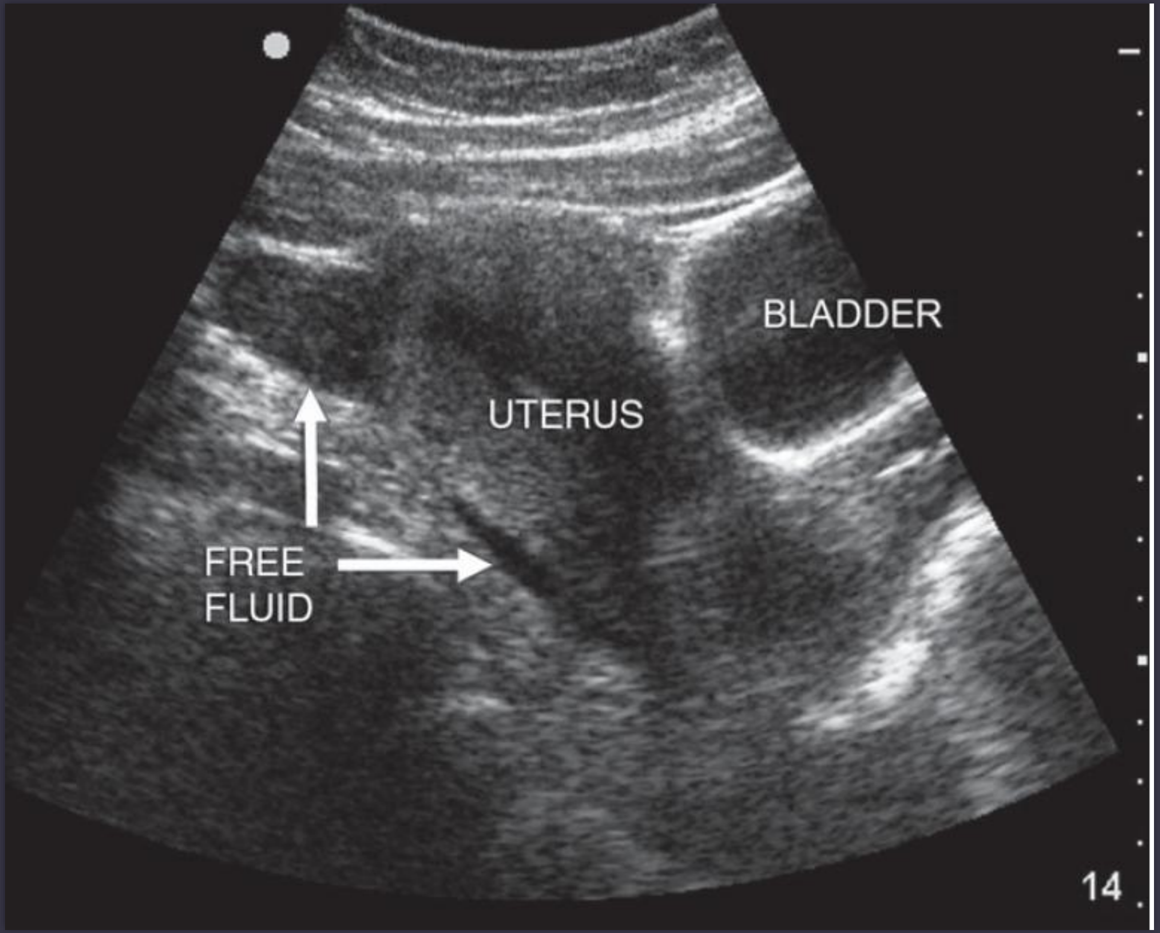
BLADDER
UTERUS
FREE
FLUID
14

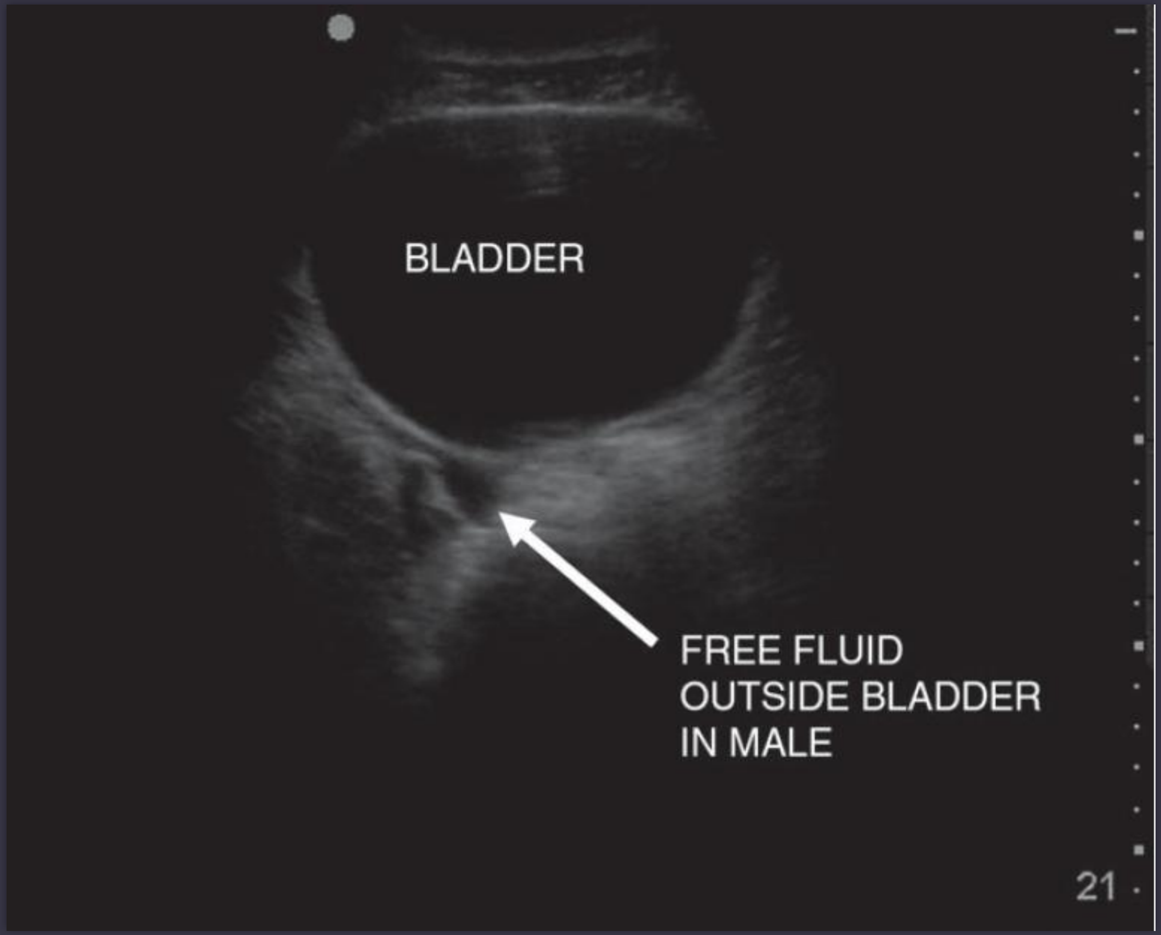
BLADDER
FREE FLUID
OUTSIDE BLADDER
IN MALE
21

Pleural effusion

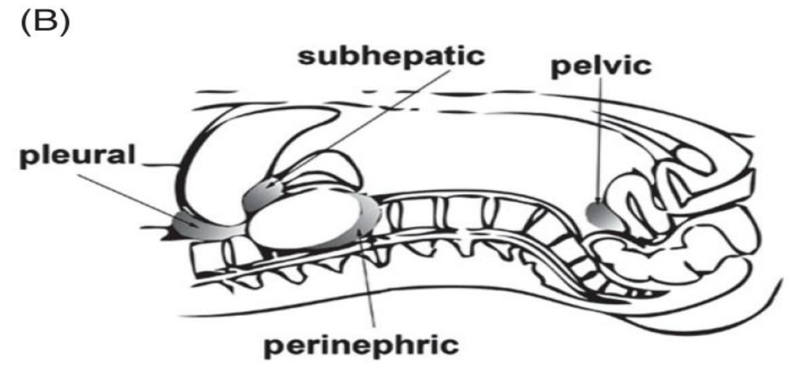

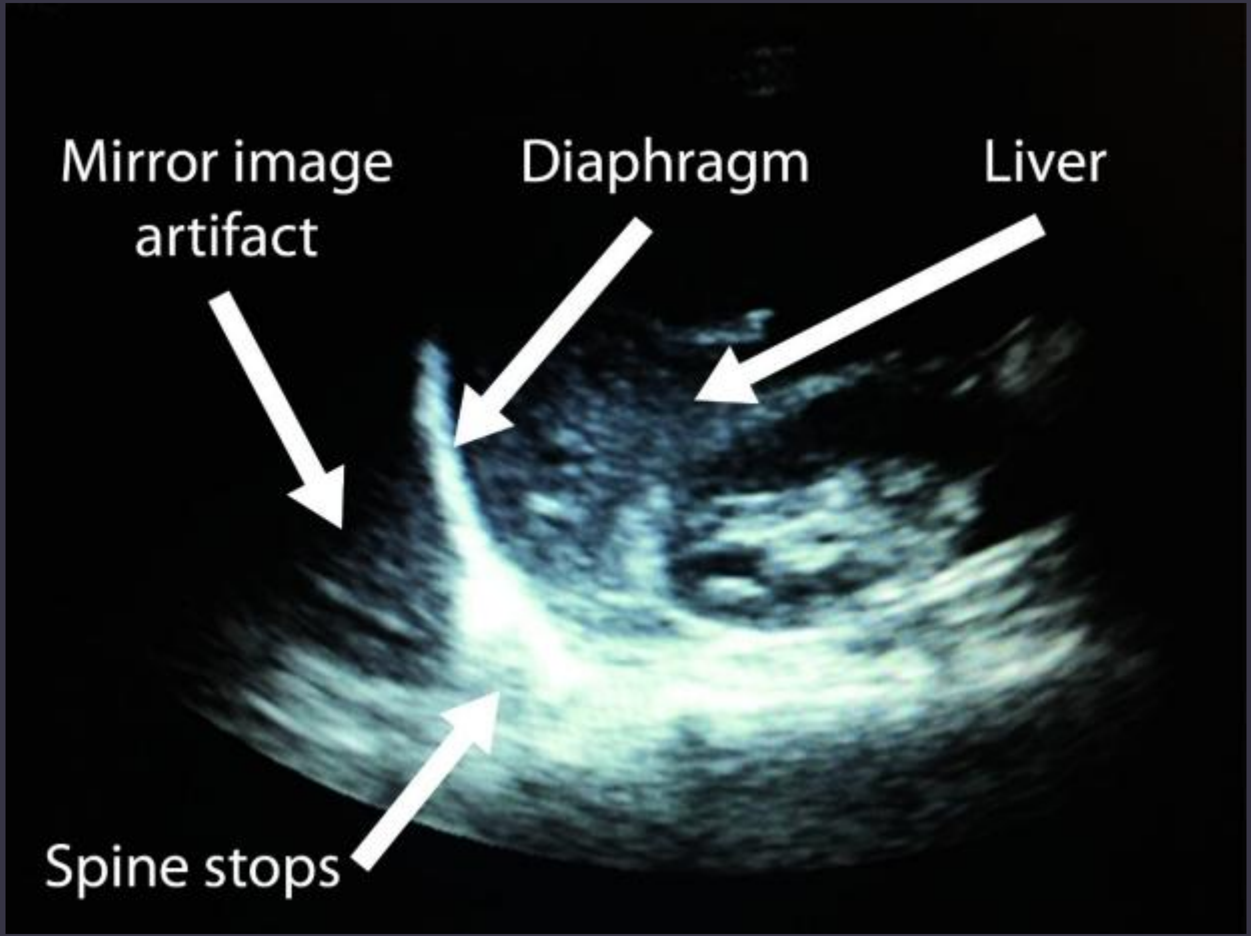

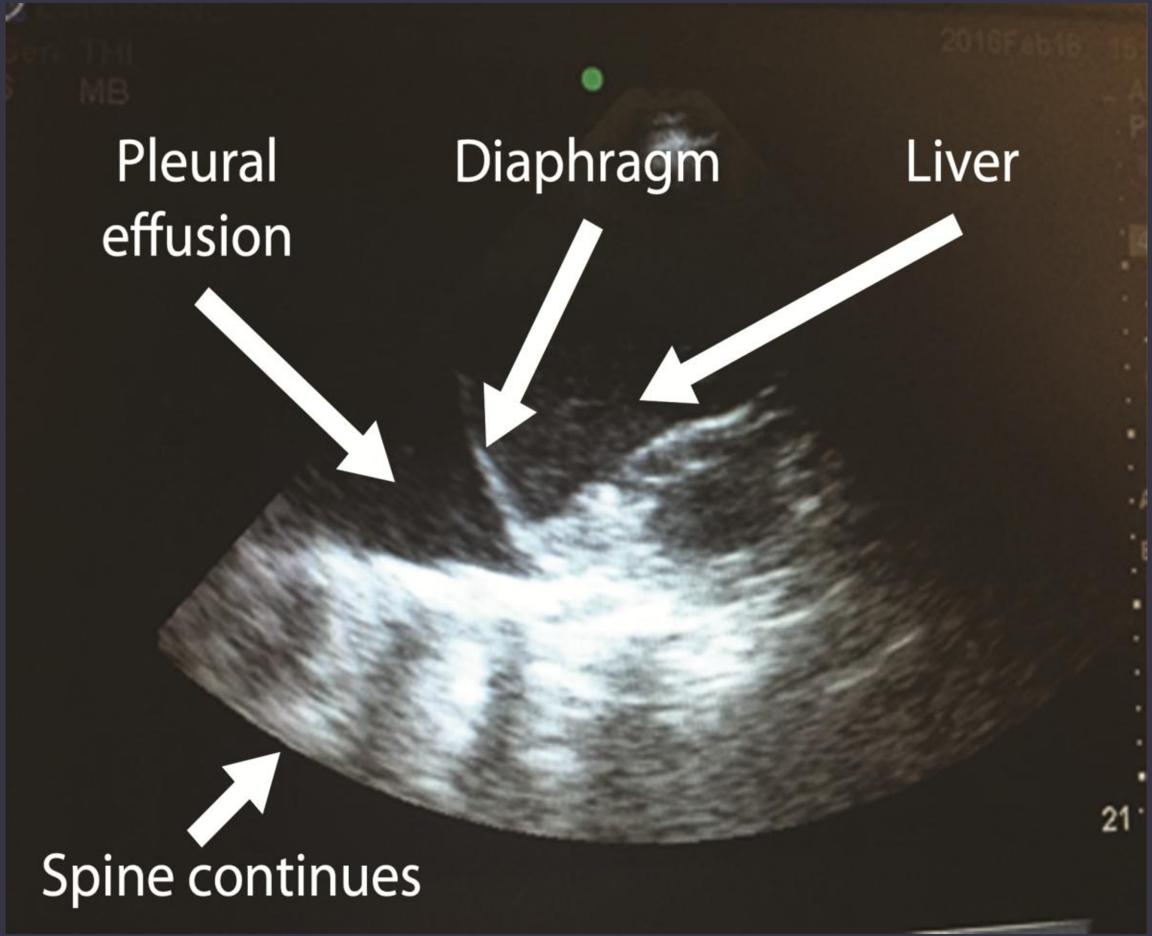

FAST Video

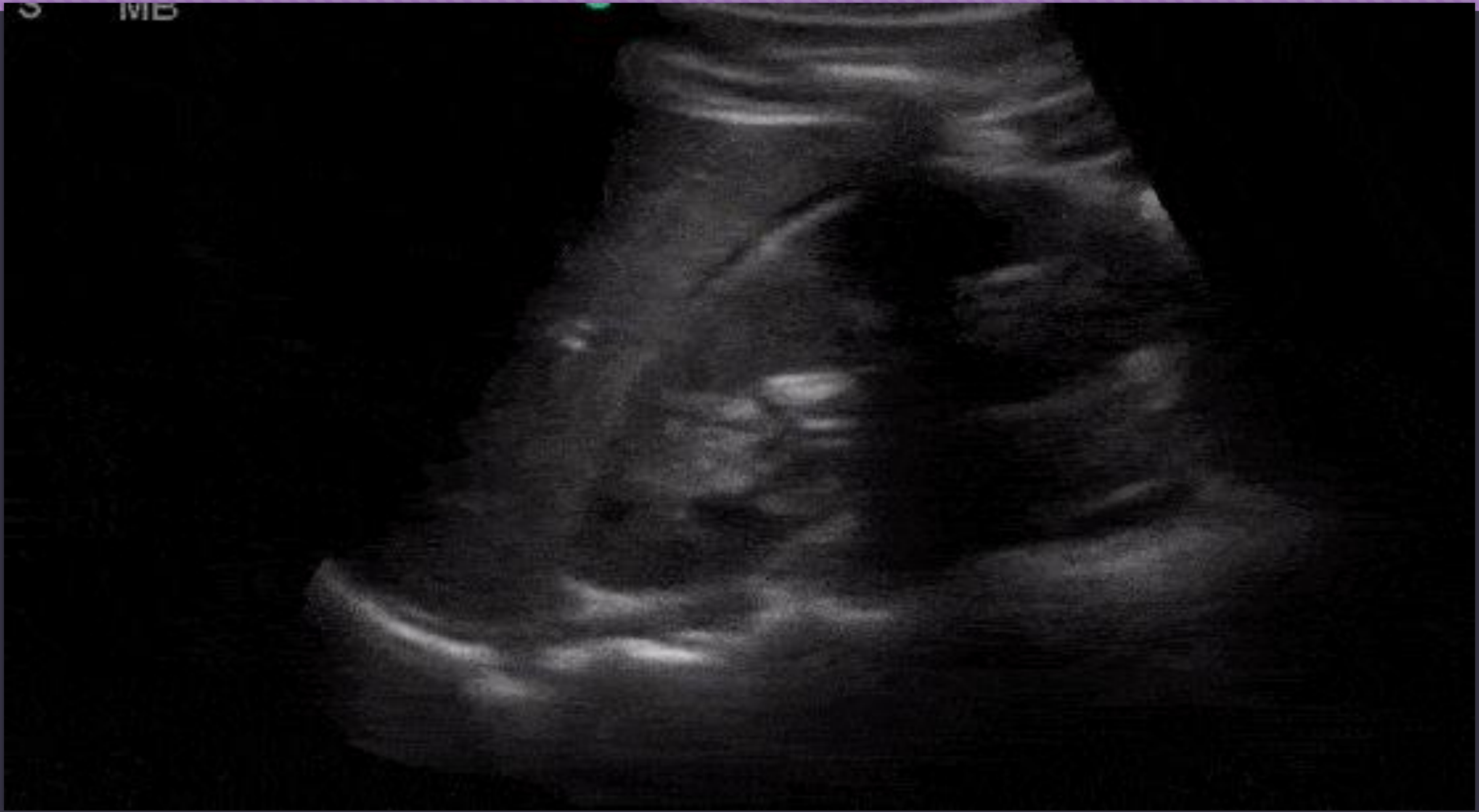

Subxiphoid cardiac view, IVC

Subxiphoid view
超音波探頭擺位

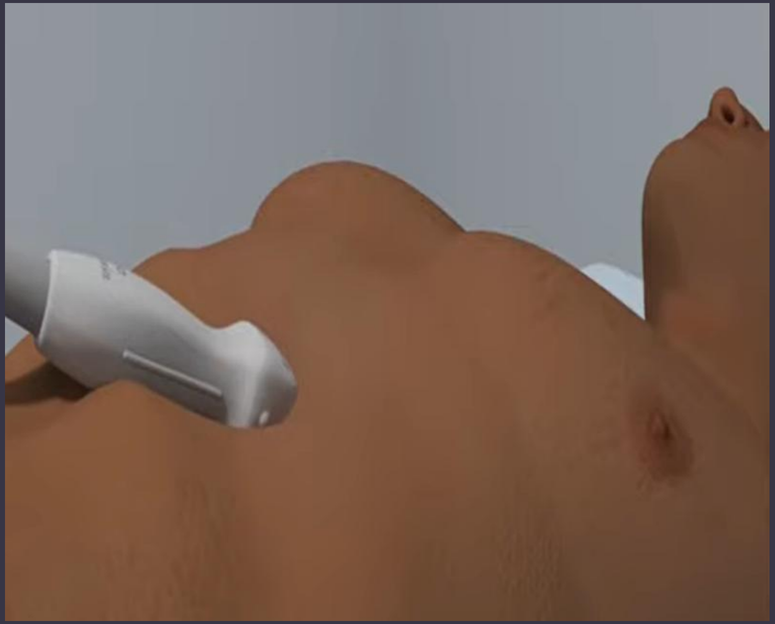

- 橫放在劍突下subxyphoid位置，一般直對頭測或些微往左肩15度 (切勿過度往左偏斜)

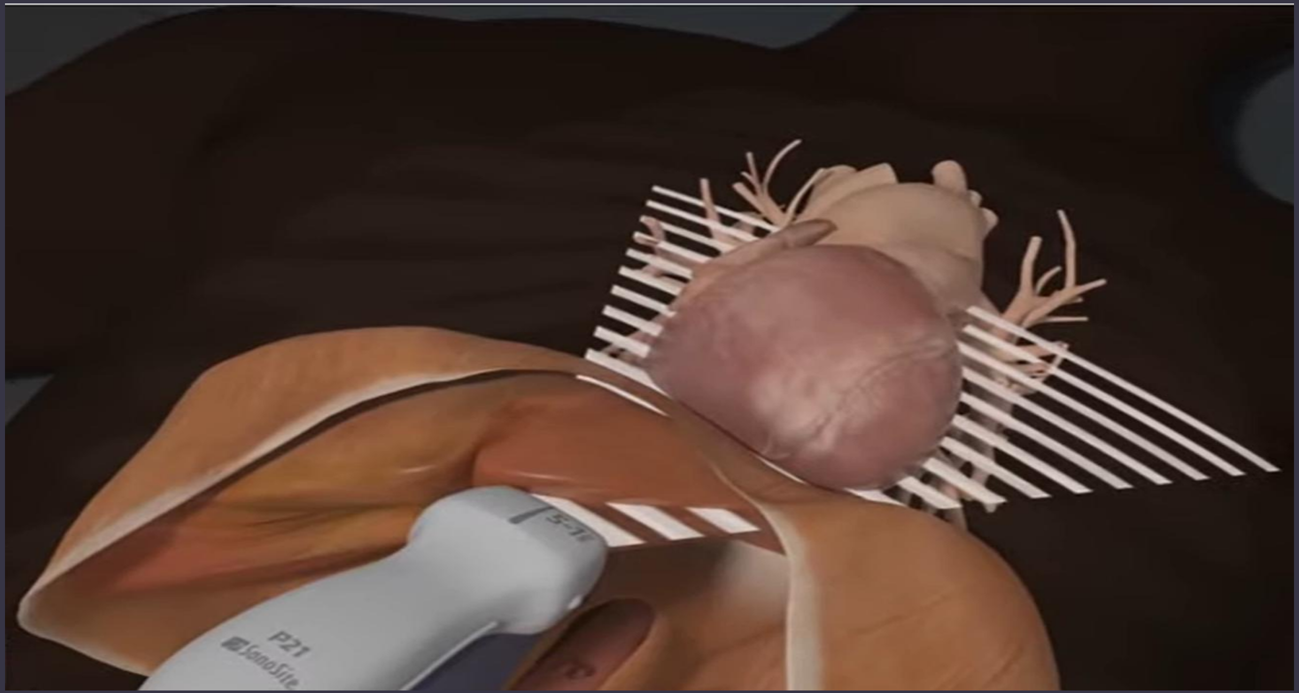

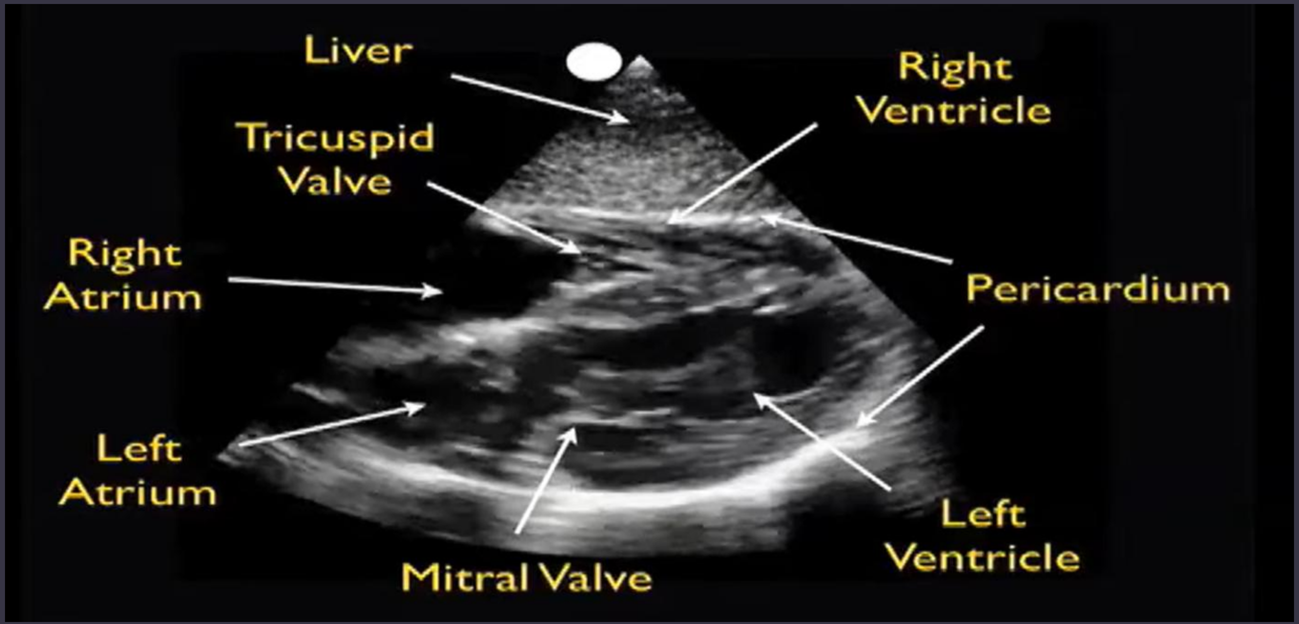

Subxiphoid view

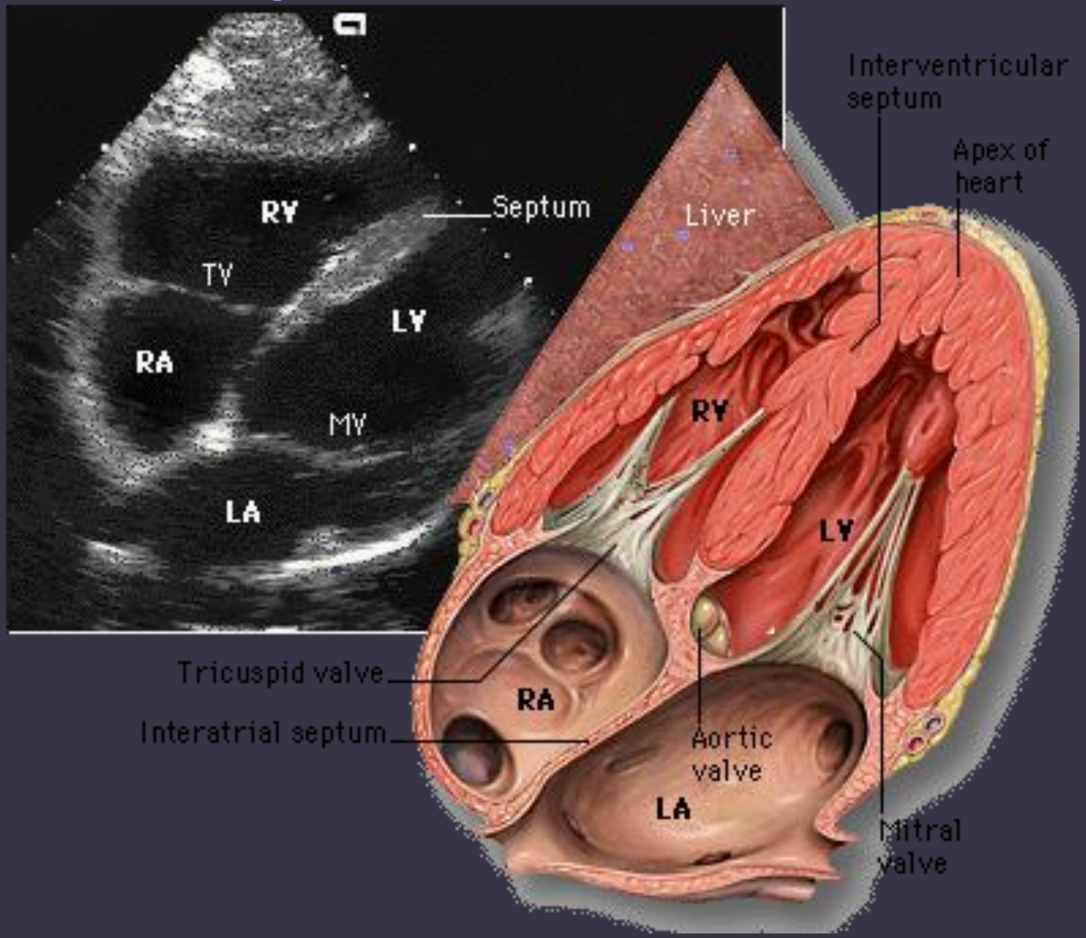

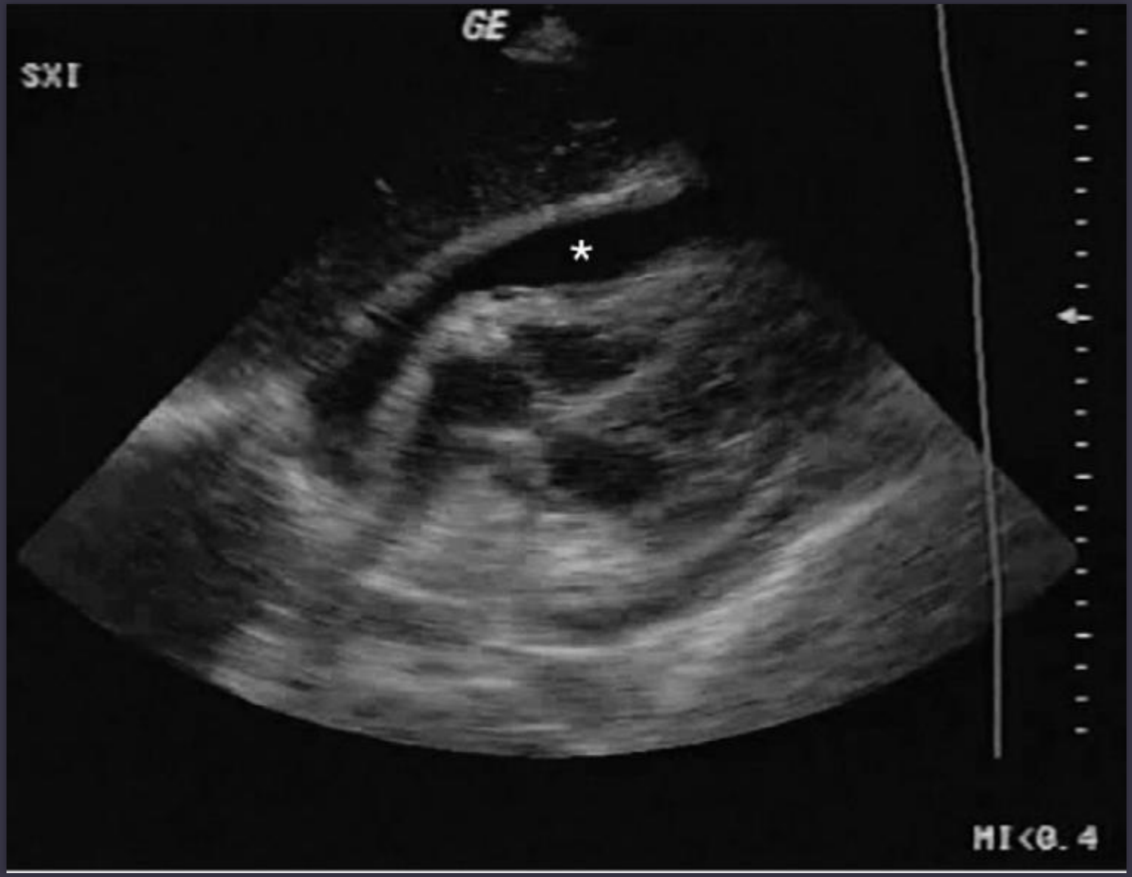

超音波掃描IVC 的臨床應用

- 評估病人血管內容積狀態(Intravascular volume status)
- 評估病人的輸液反應性(fluid responsiveness)
- 評估是否有心包膜填塞(cardiac tamponade)
- 評估是否有栓塞(IVC thrombus)

IVC diameter, respiratory variation and RA pressure

IVC (cm)	Respiratory change	RA pressure (cm H_2O)
< 1.5	Total collapse	0–5
1.5–2.5	$>$ 50% collapse	5–10
1.5–2.5	$<$ 50% collapse	11–15
> 2.5	$<$ 50% collapse	16–20
> 2.5	No change	> 20
Reproduced with permission from Wong & Otto 2000 [1].		

Caval Index = (IVC exp diameter - IVC insp diameter) / (IVC exp diameter) * 100

測量IVC之呼吸變異的位置

- 距離 IVC 注入 RA 交接口處(RA-IVC junction)約 2cm
- 肝靜脈(hepatic vein) 匯集注入IVC 開口處

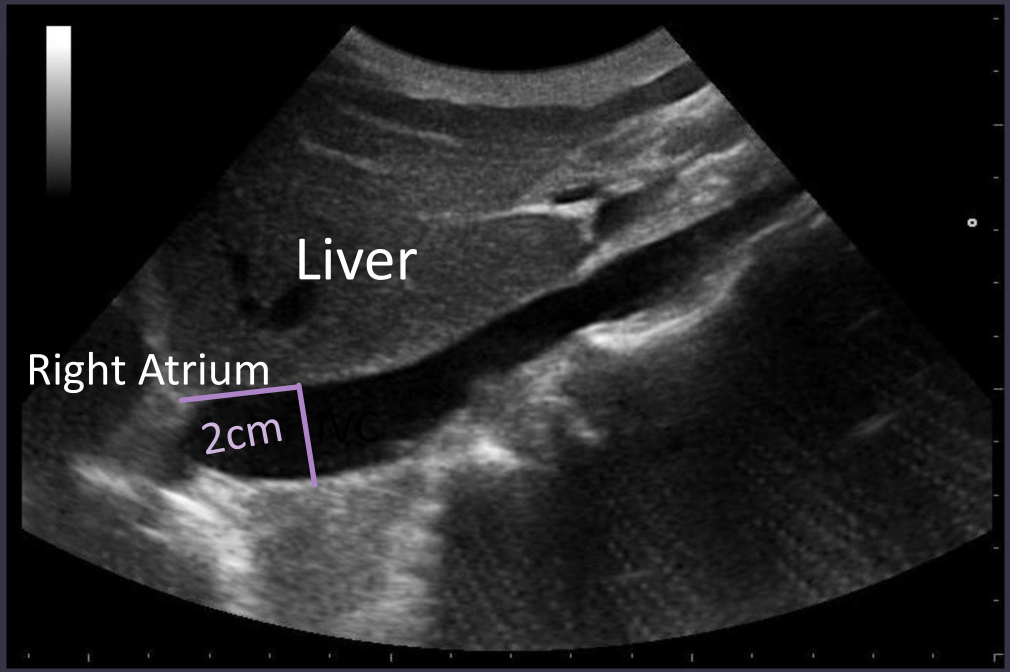

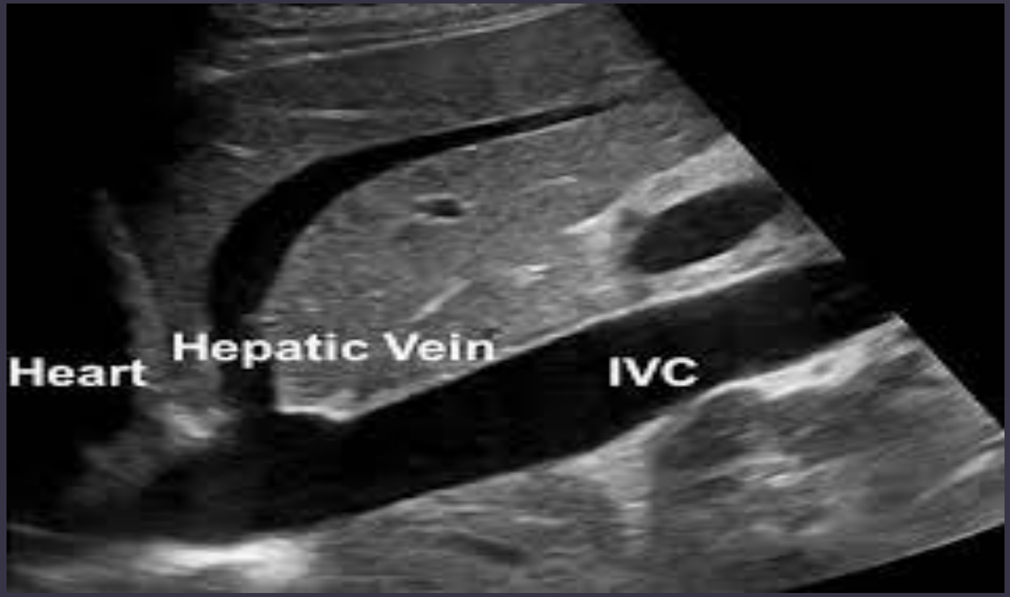

M Mode

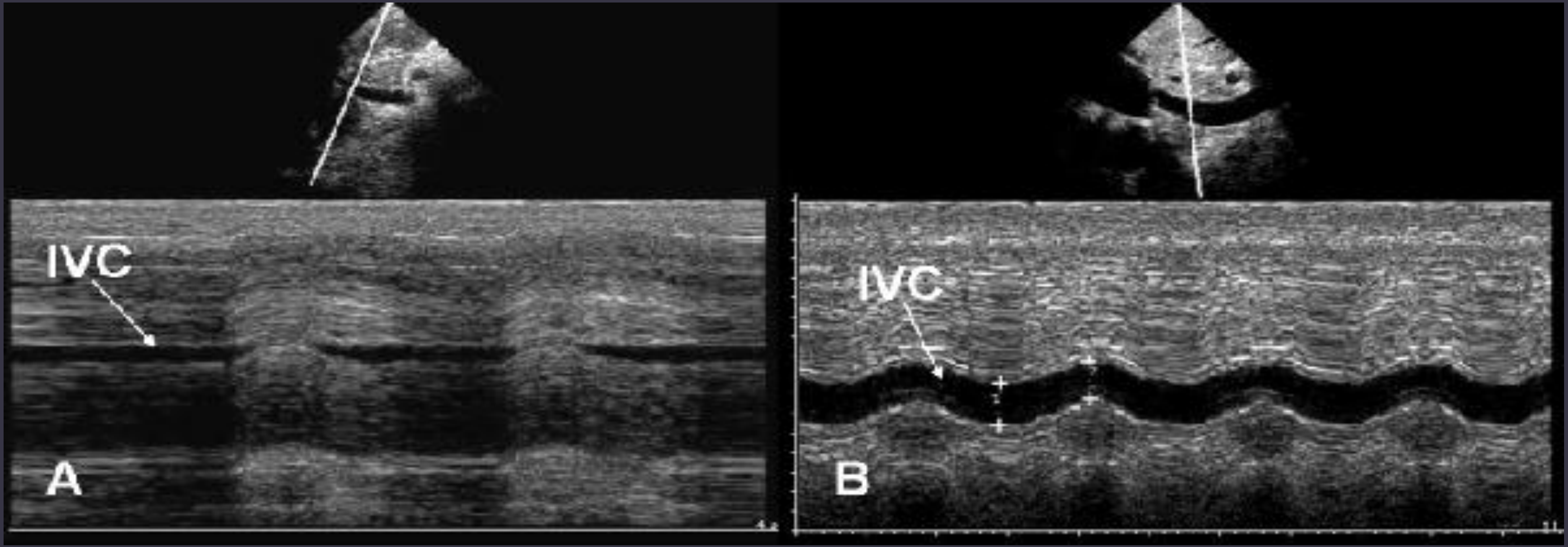

Abdomen: liver, GB, kidney, UB

Longitudinal scan

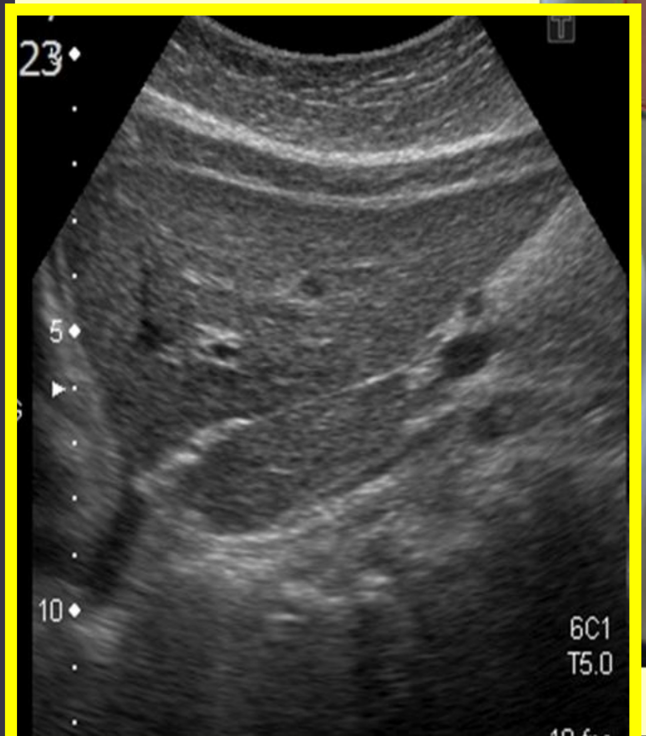

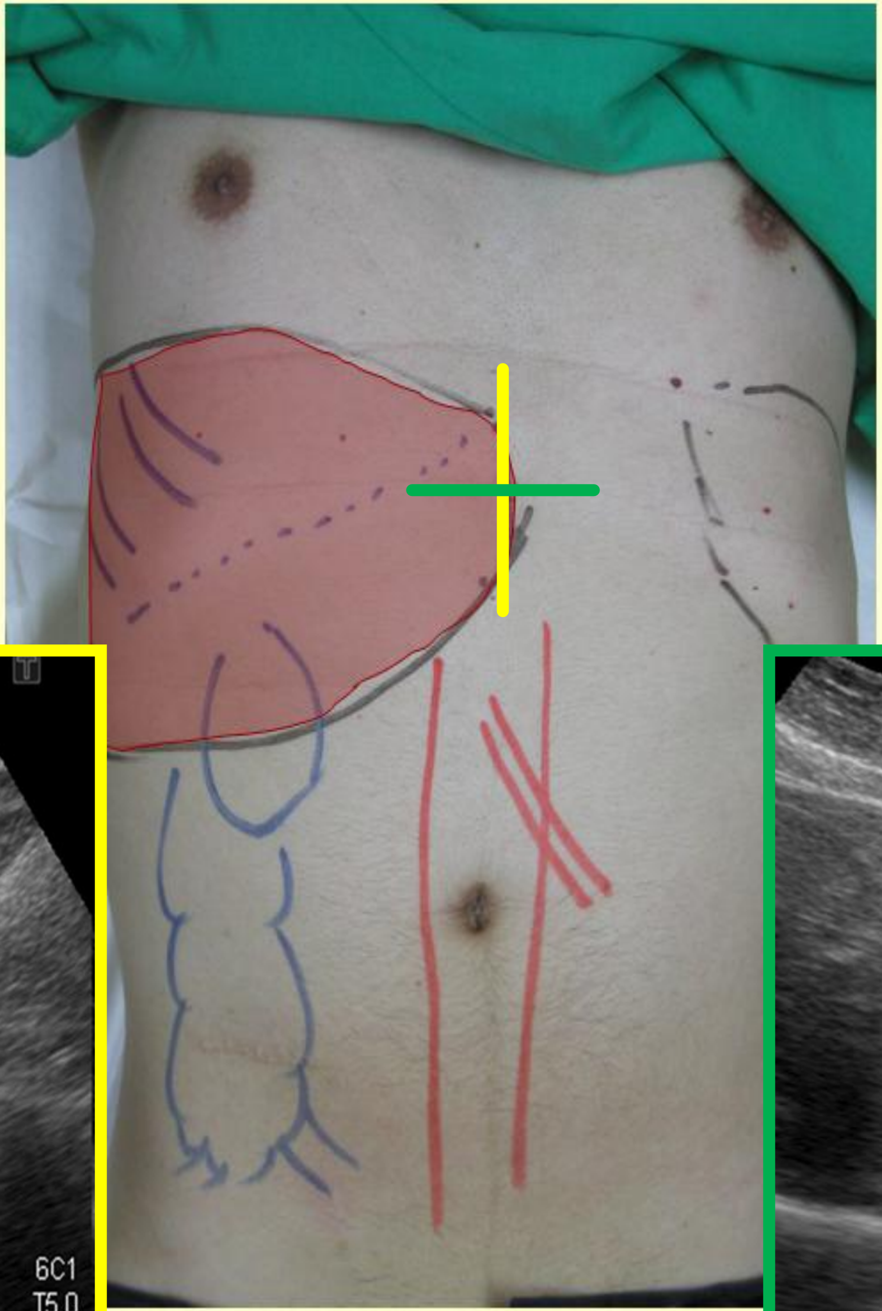

Transverse scan

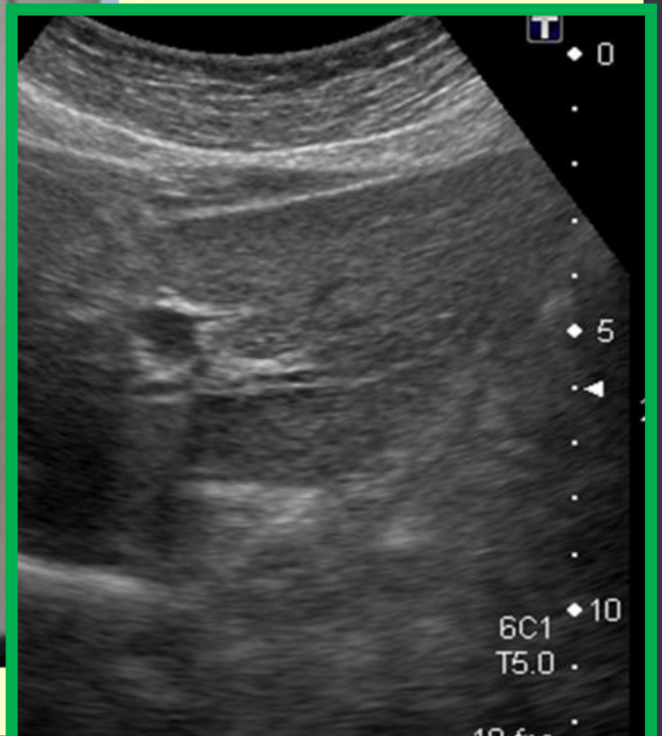

Subcostal (肋骨下緣) view

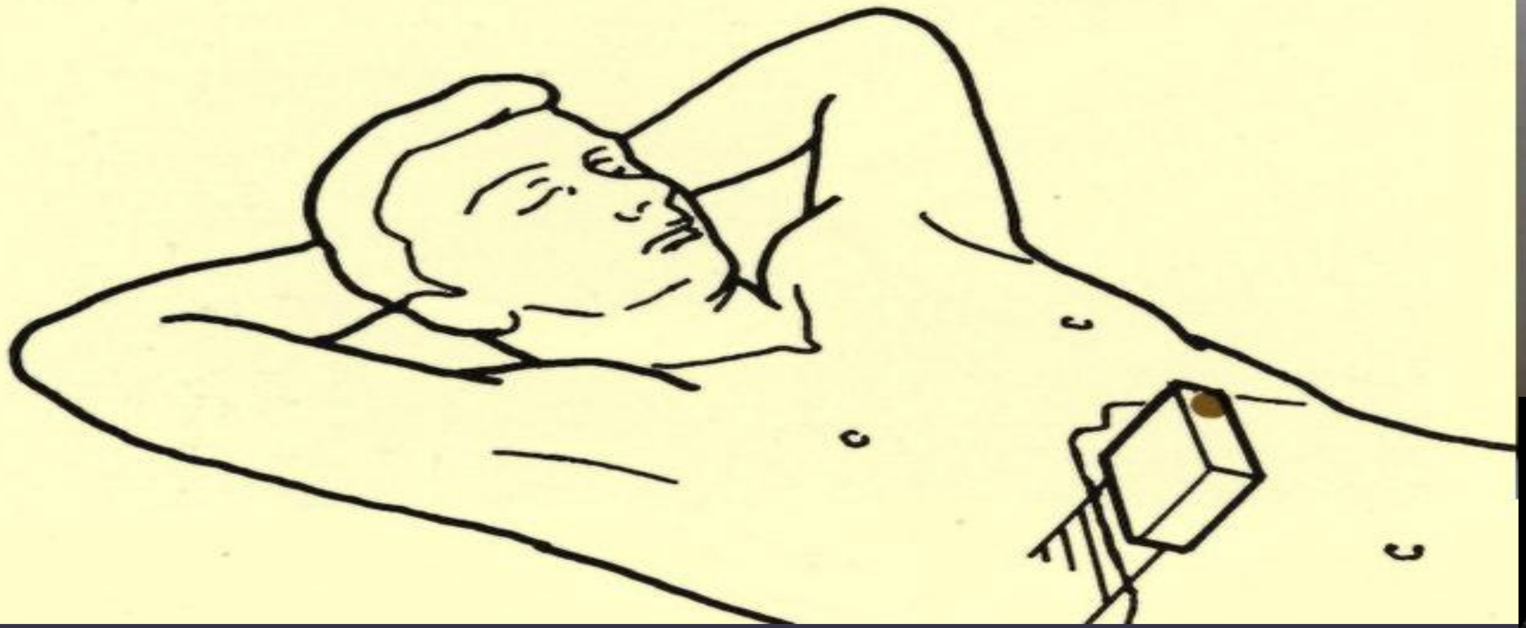

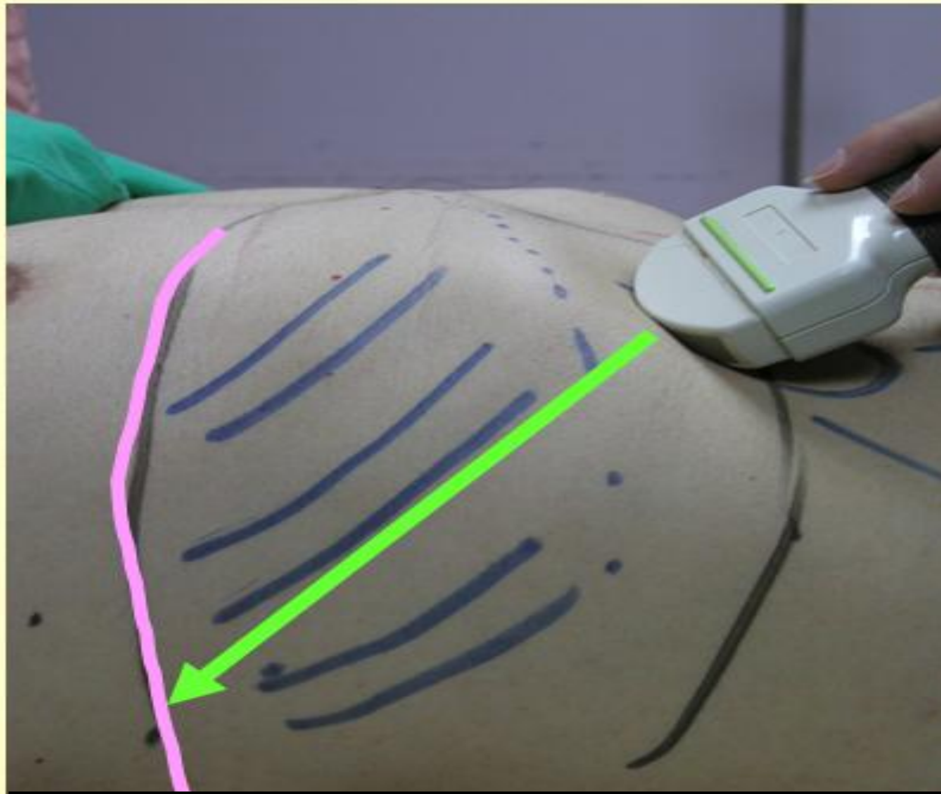

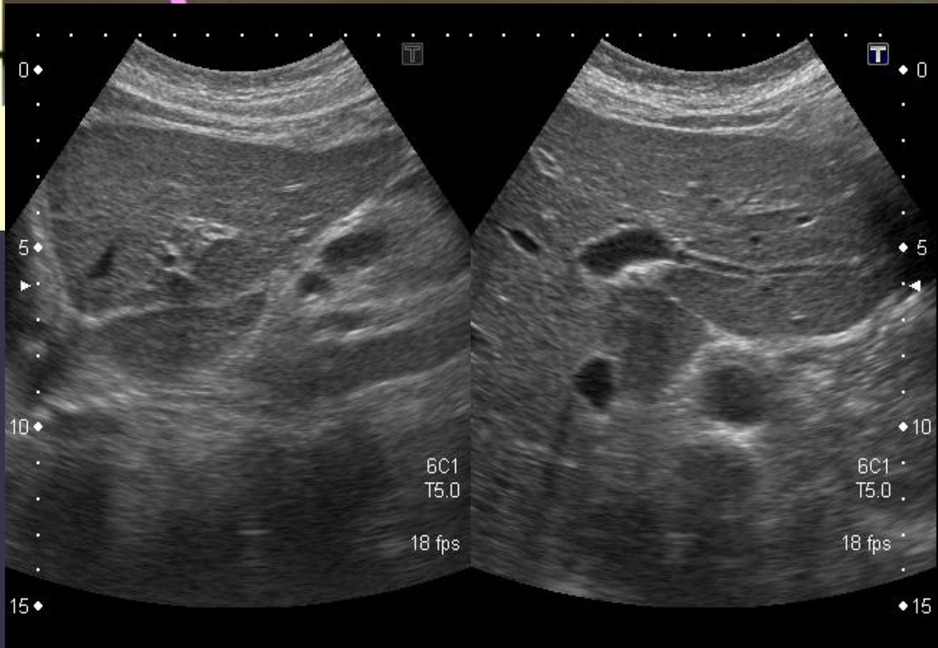

Intercostal (肋骨間隙) view

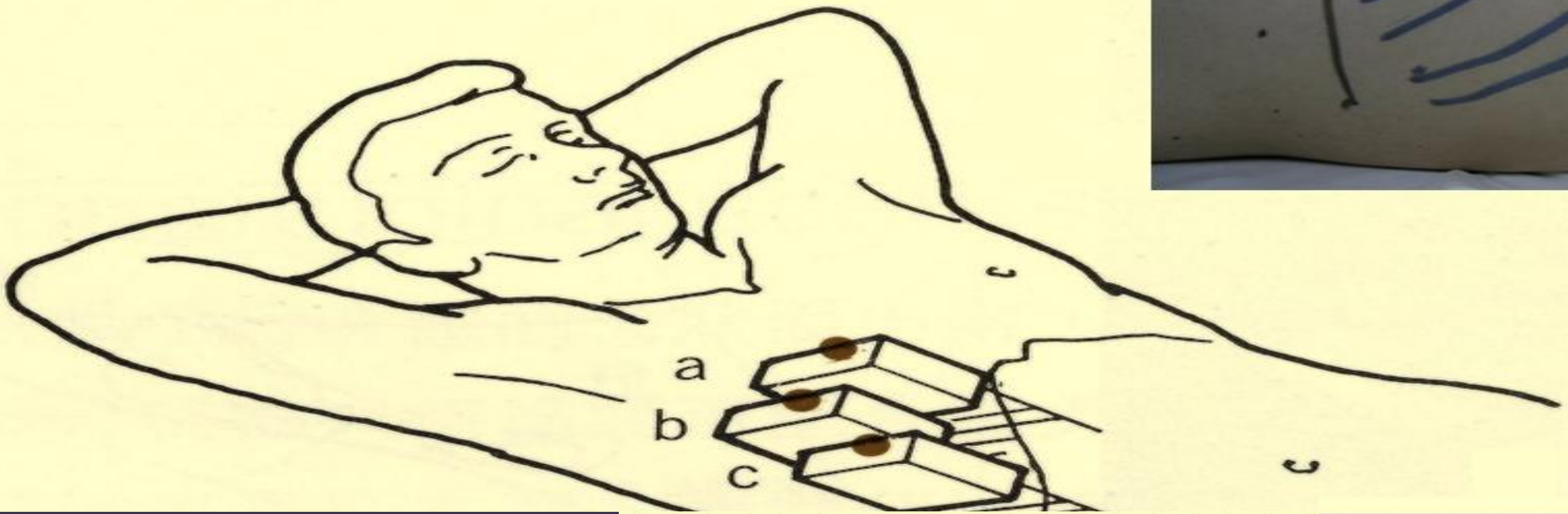

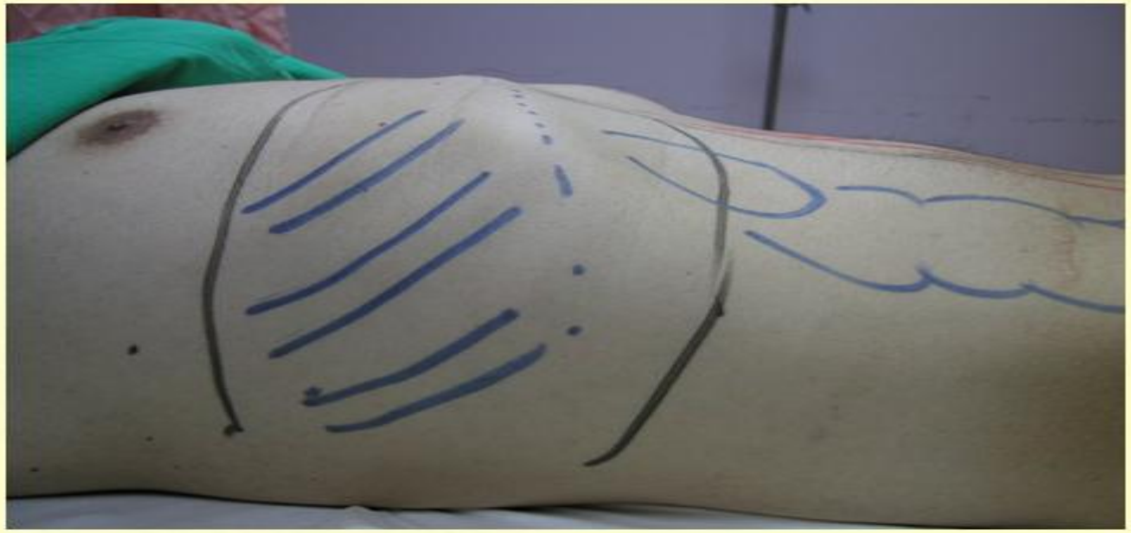

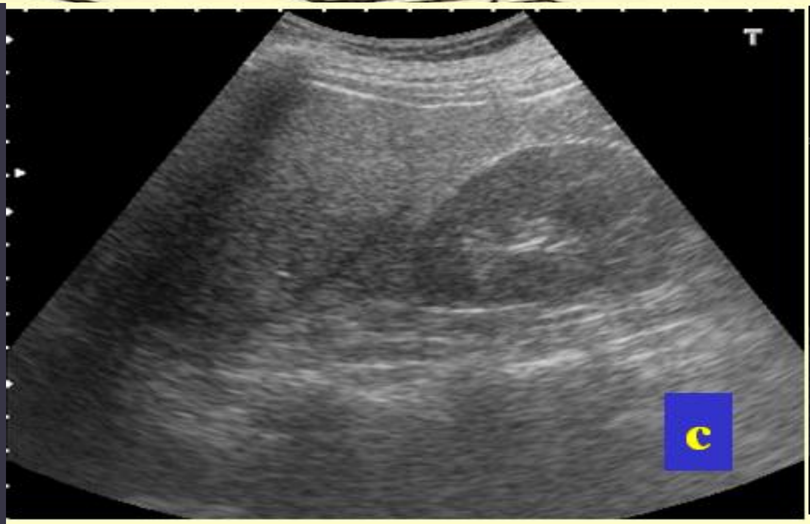

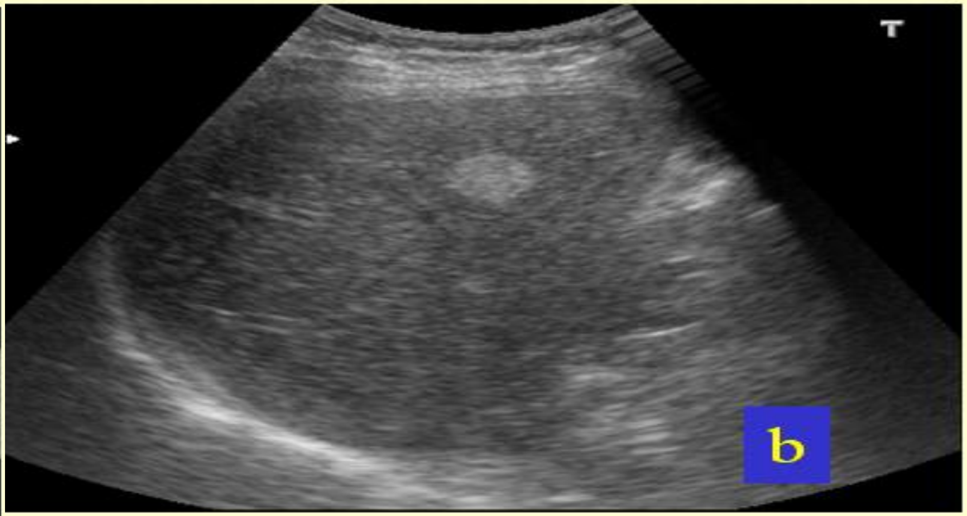

膽囊 (Gallbladder)

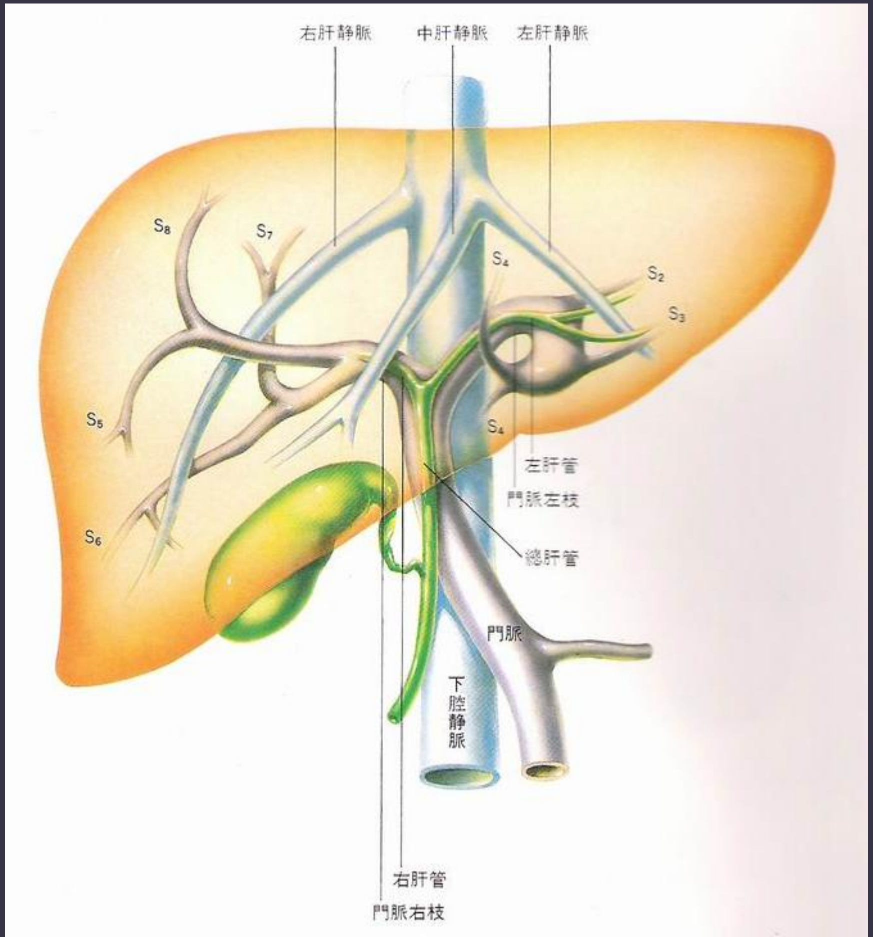

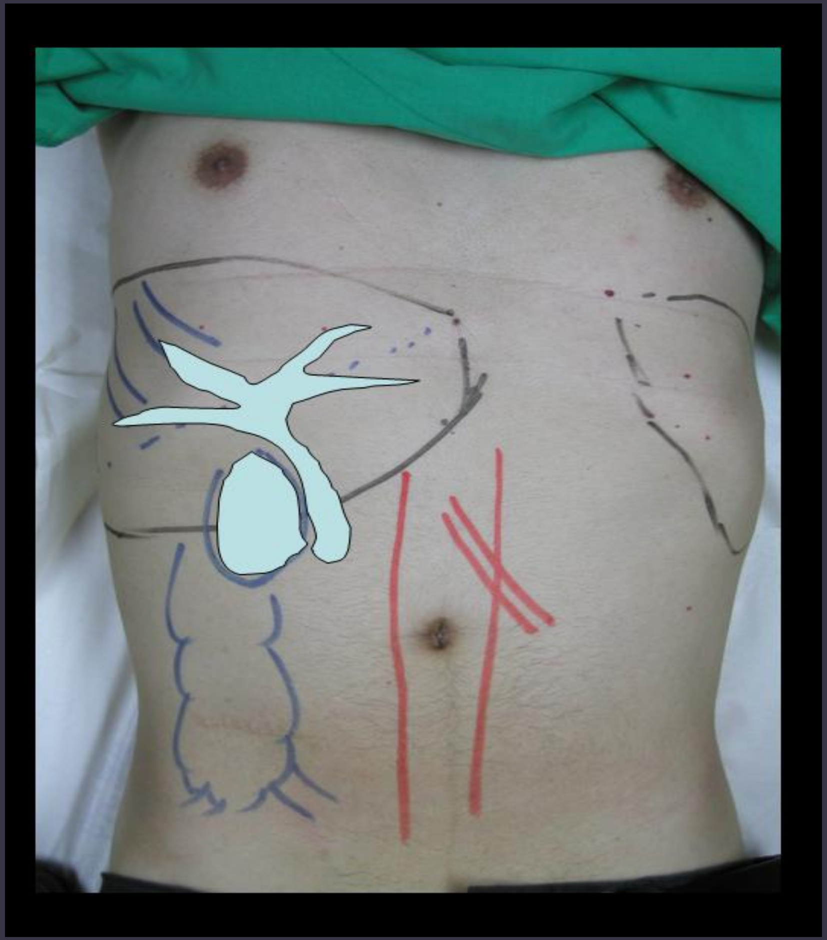

Gall Stone

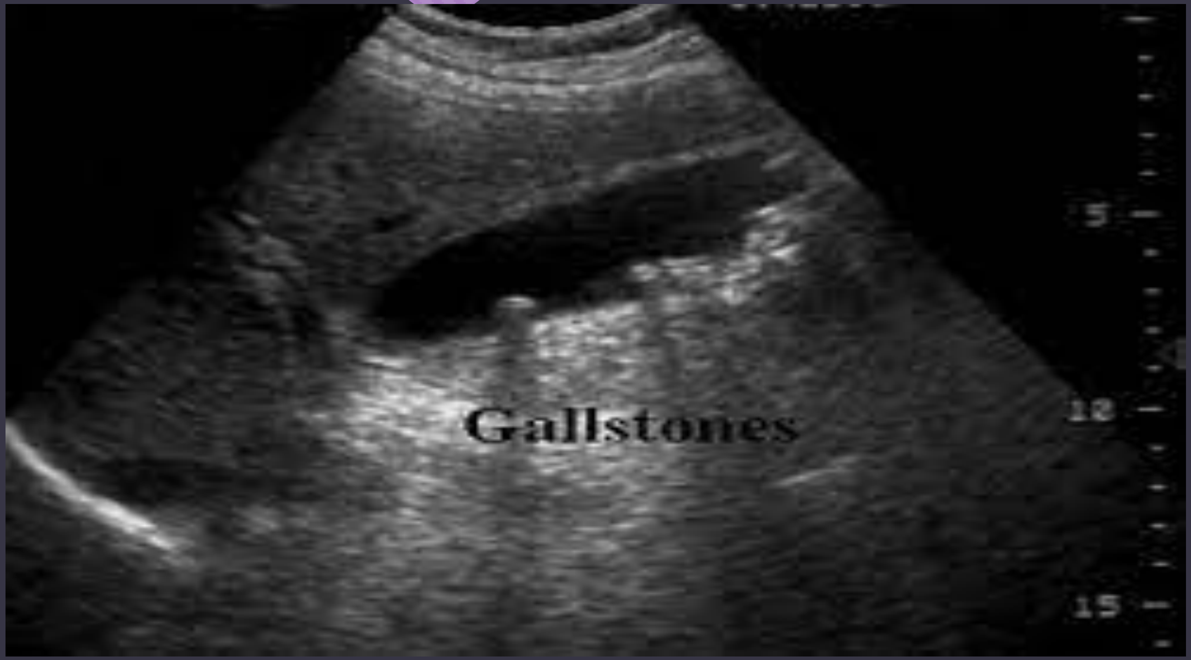

- Hyperechoic lesion with posterior acoustic shadows

GB polyps

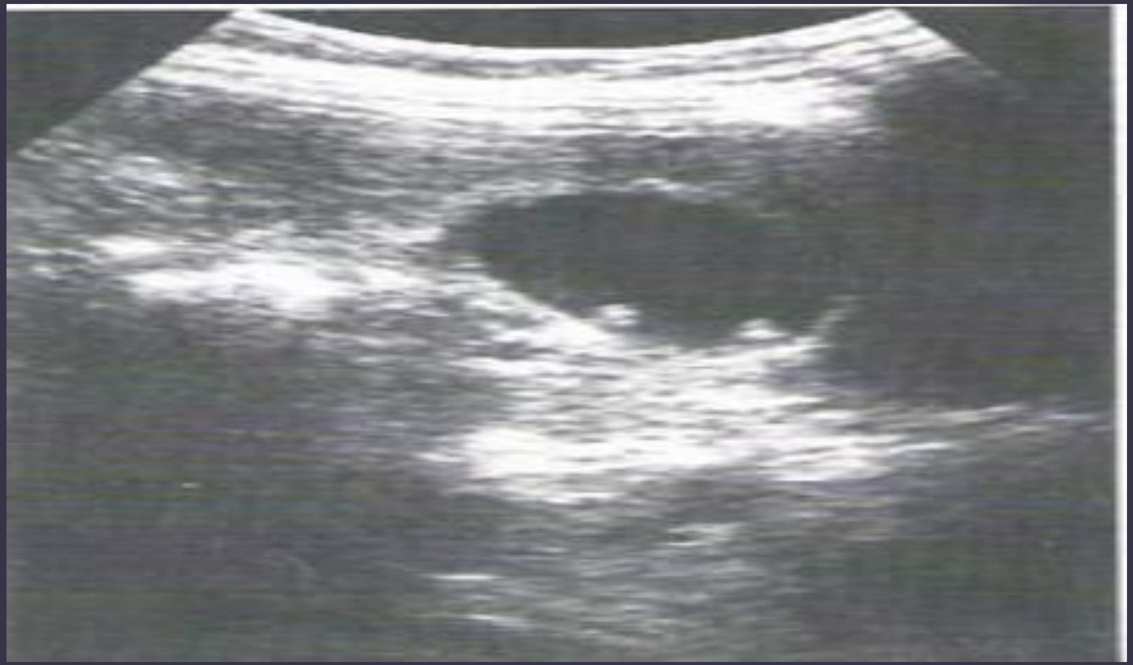

- No acoustic shadows
- Not movable during changing position

Acute cholecytitis

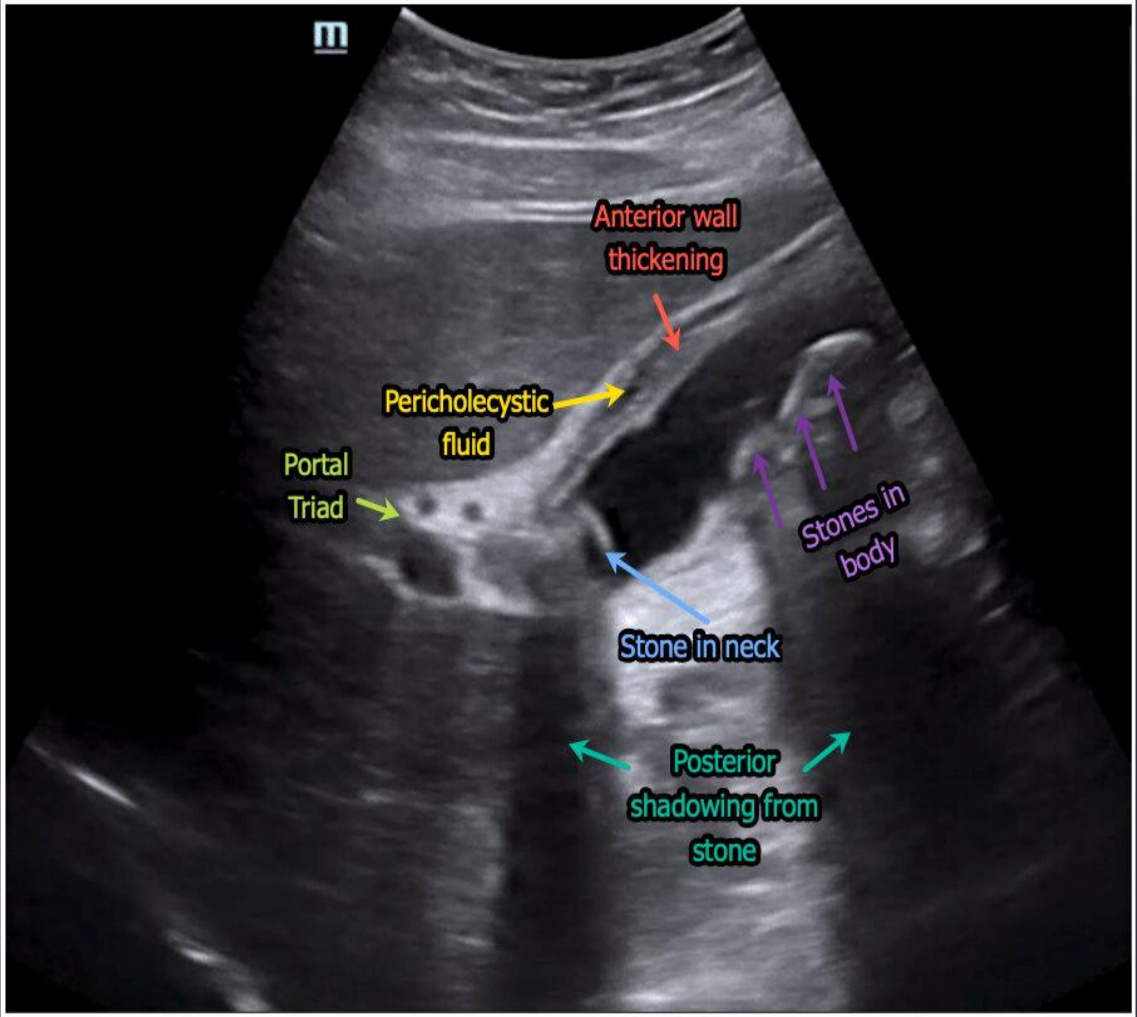

The most sensitive US finding in acute cholecystitis is the presence of cholelithiasis in combination with the **sonographic Murphy sign.**

Others are secondary findings.

- **Ultrasonographic Murphy's sign**
- Distended GB
- gallbladder wall thickening (>3 mm)
- Wall layering
- Peri-GB fluid

腎臟超音波簡介

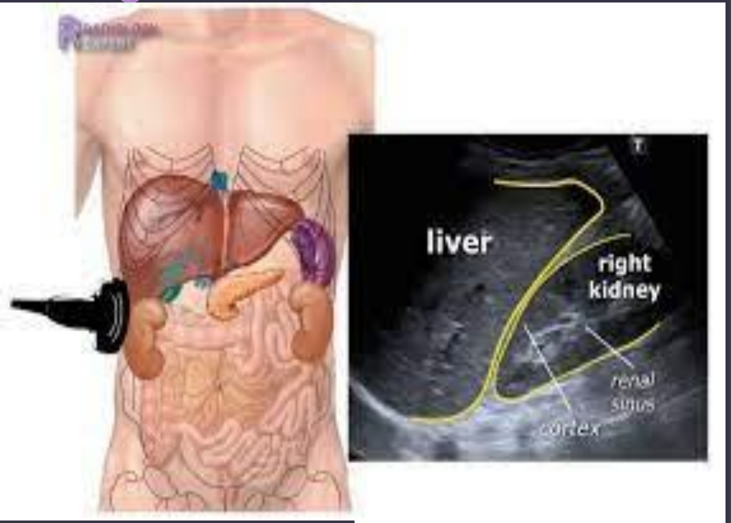
liver
right kidney
renal sinus
cortex

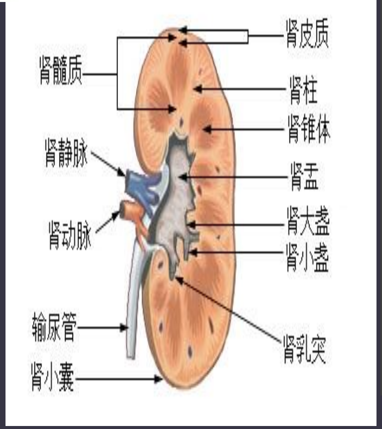
肾皮质
肾髓质
肾柱
肾锥体
肾静脉
肾盂
肾大盏
肾动脉
肾小盏
输尿管
肾乳突
肾小囊

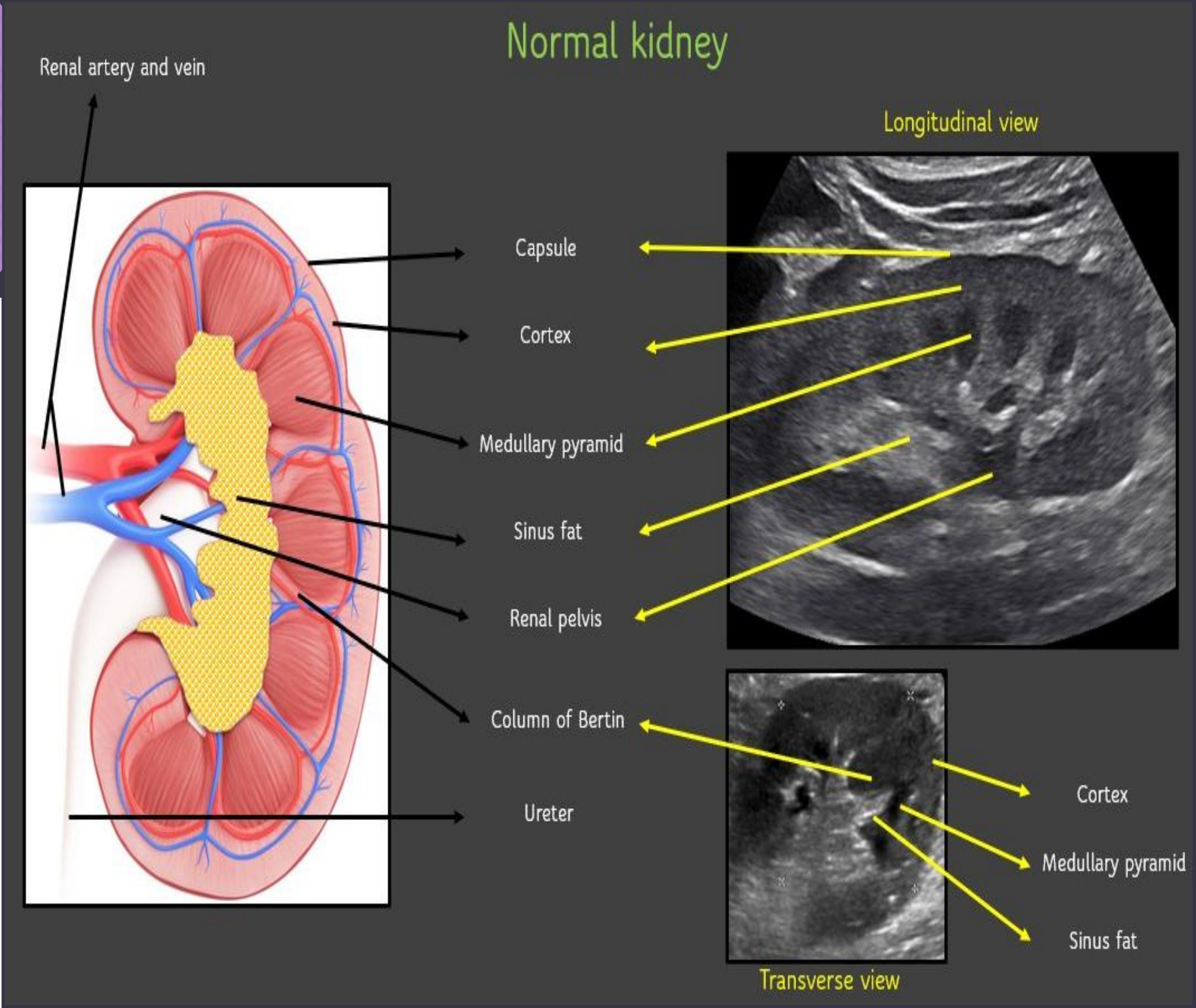
Normal kidney
Renal artery and vein
Longitudinal view
Capsule
Cortex
Medullary pyramid
Sinus fat
Renal pelvis
Column of Bertin
Ureter
Cortex
Medullary pyramid
Sinus fat
Transverse view

腎臟超音波簡介

- 9–12 cm in length
- 4–5 cm in width
- Within 2 cm of each other in terms of size

Hydronephrosis

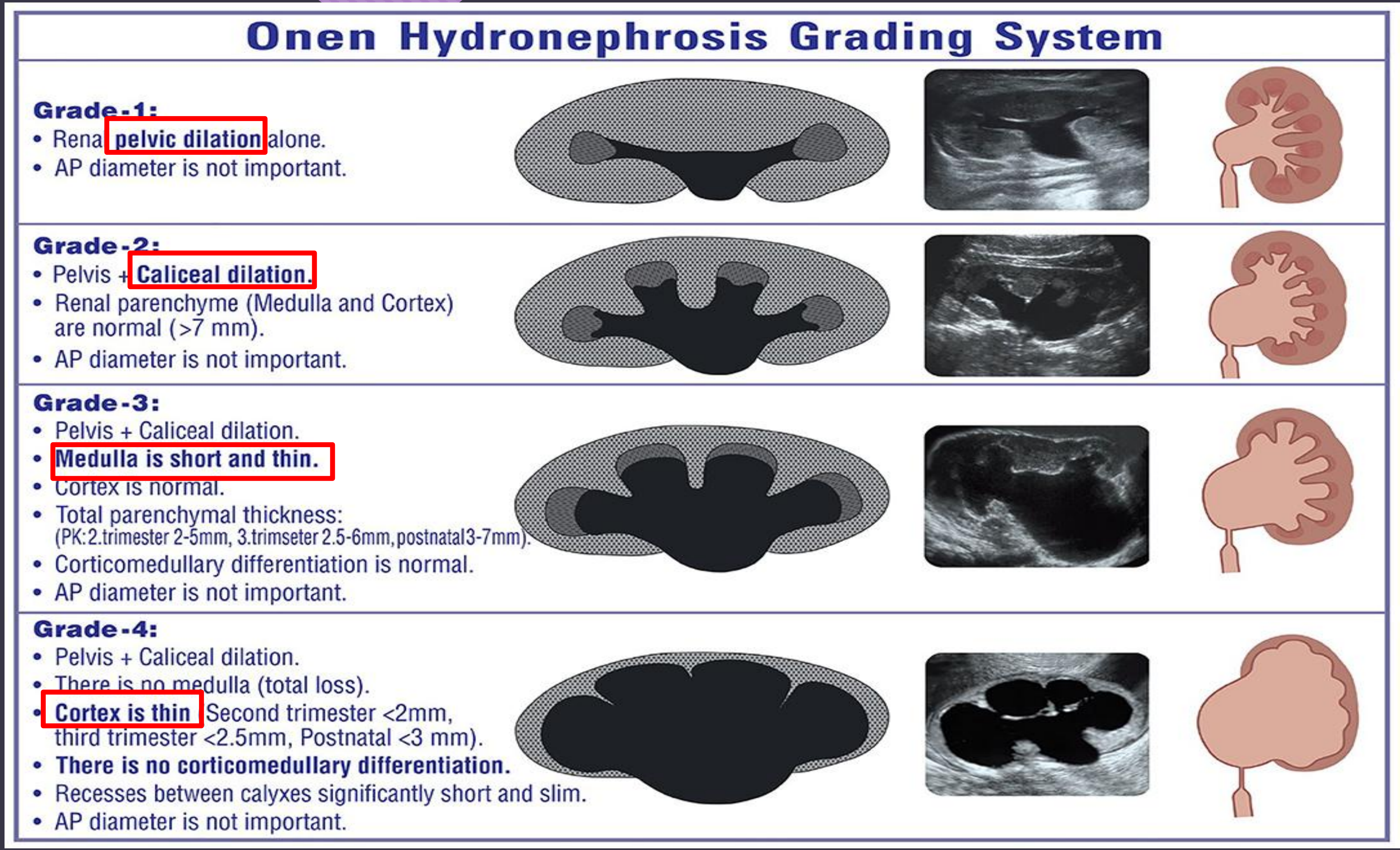

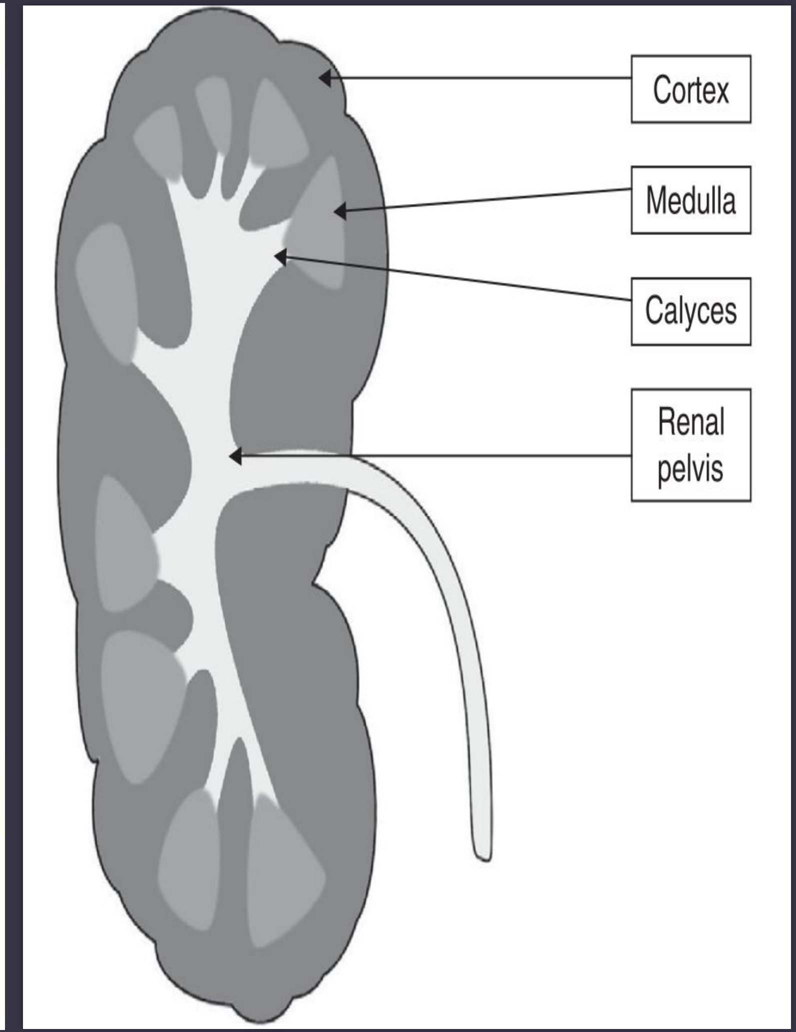

Bladder volume estimation

0.75 * width* length *height VS (0.52*width* length *height)

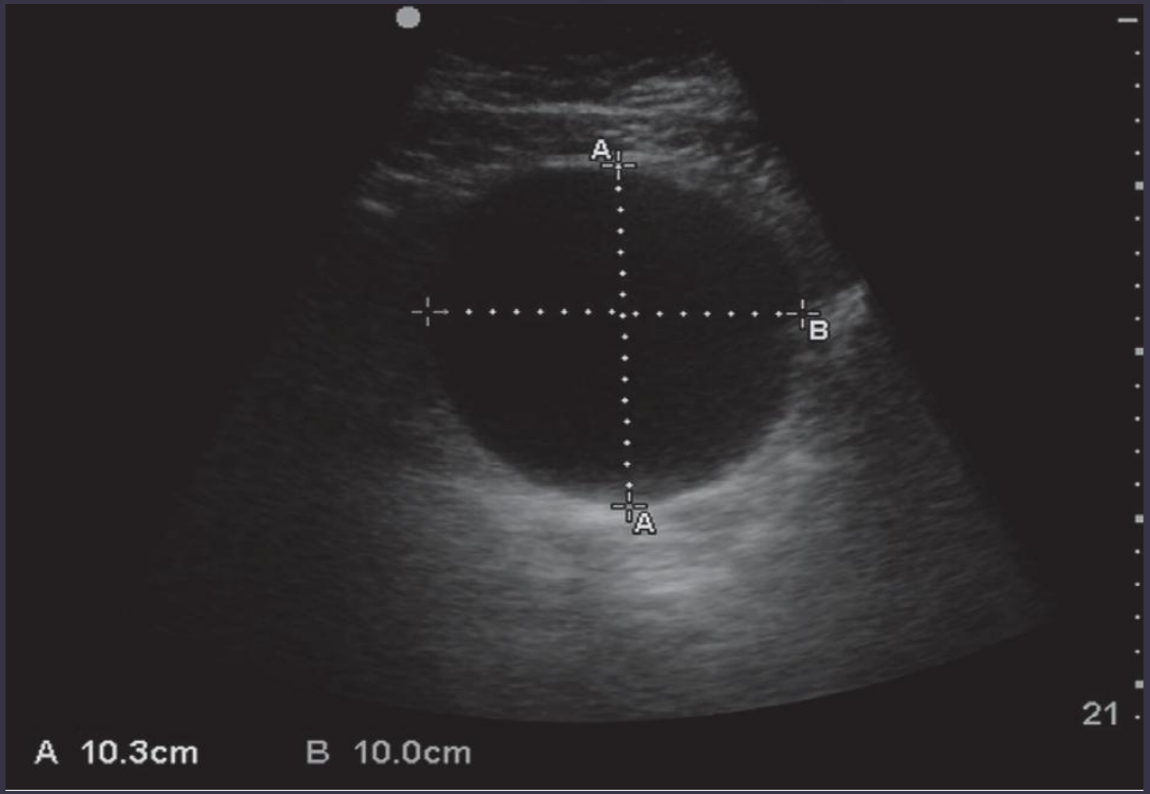

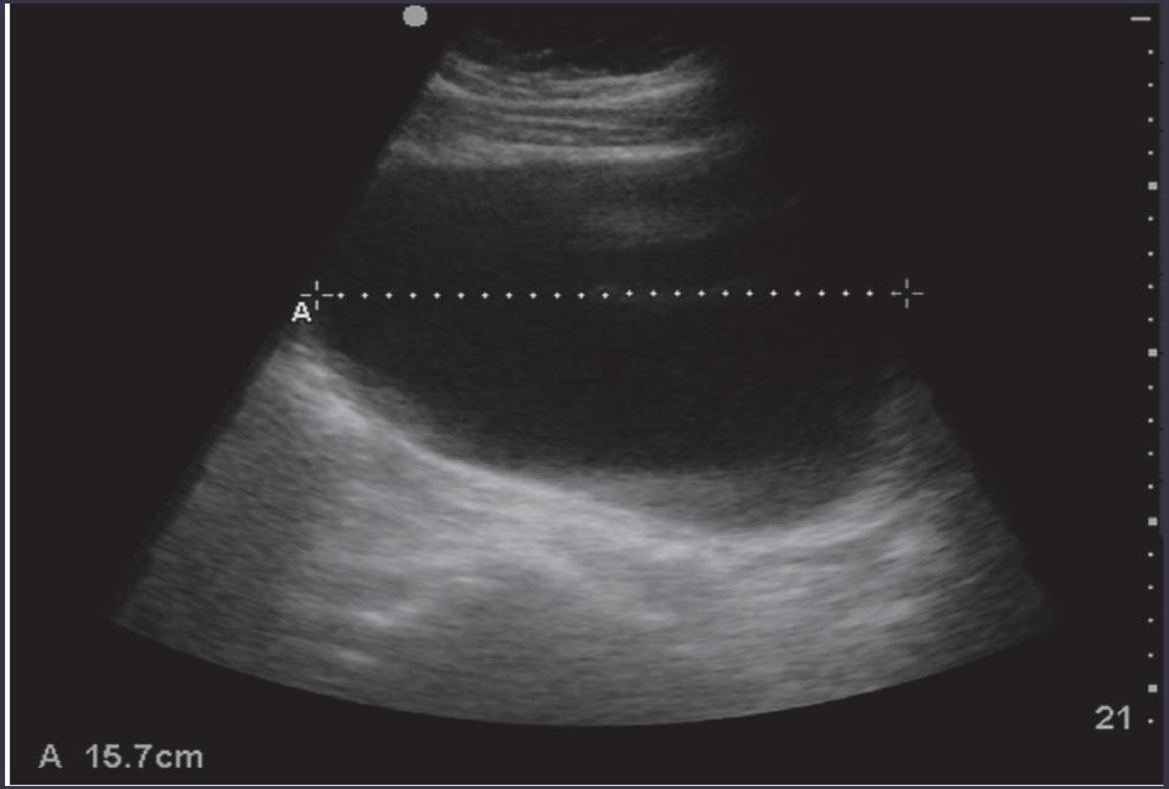

Venous cannulation

Venous cannulation常用術語介紹

- 靜態(Static): ultrasound-assissted
- 即時(Real-time): direct, ultrasound-guided
- 短軸(Short-axis): cross section
- 長軸(Long-axis): longitudinal
- 平面內(In-plane):針頭平面與超音波平面相同，無法看到進針狀況
- 平面外(Out-of-plane)：針頭平面與超音波平面不同，整個過程均可見到針頭

In-plane & out-of-plane

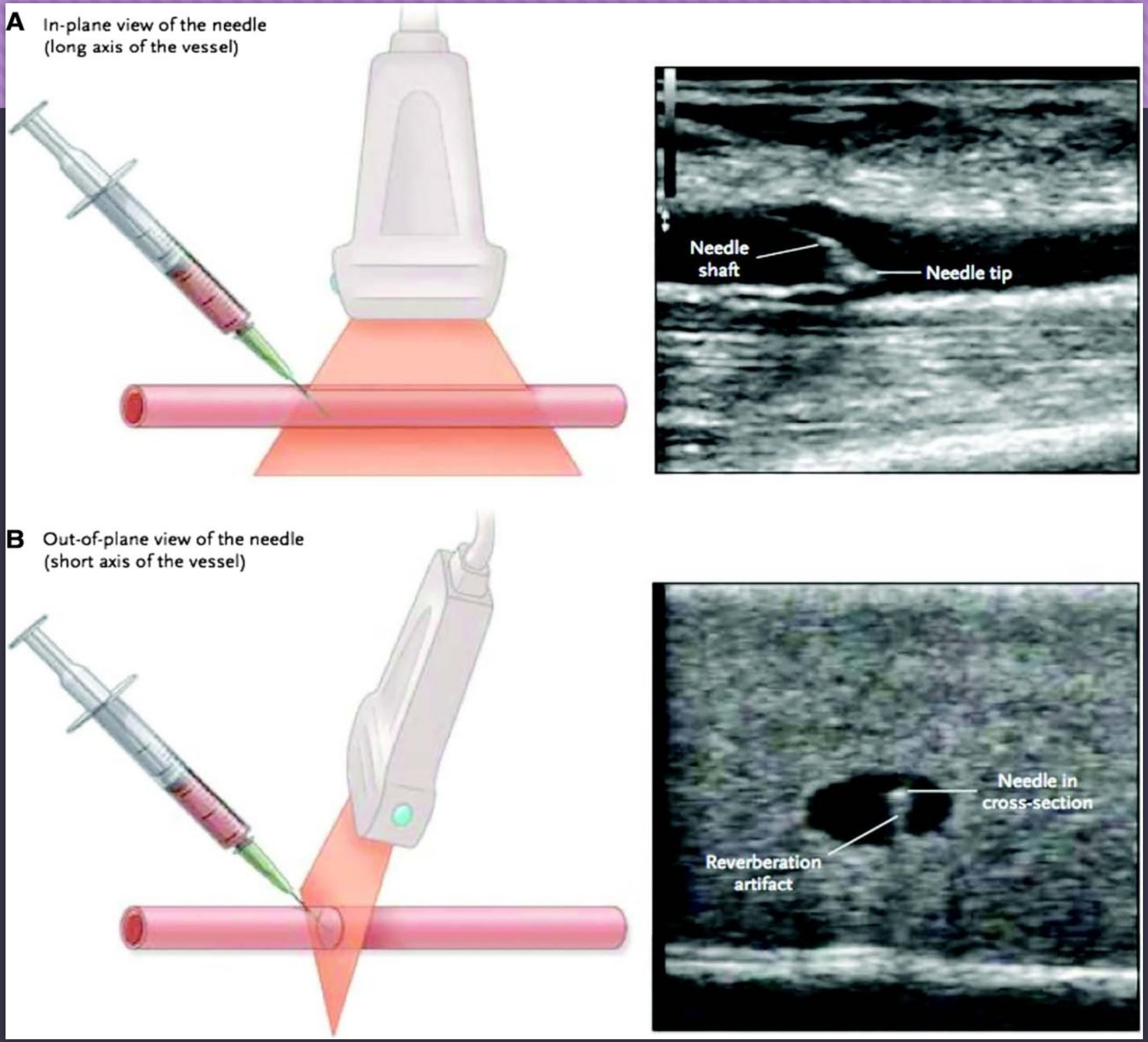

J of Ultrasound Medicine, Volume: 38, Issue: 3, Pages: E4-E18, First published: 13 February 2019, DOI: (10.1002/jum.14954)
